# Catalytic Ammonia Synthesis over Pure, Defective, and Metal-Doped Rutile TiO_2_: A Periodic DFT Study

**DOI:** 10.1021/acs.jpcc.5c06031

**Published:** 2026-01-27

**Authors:** Francisco Núñez-Zarur, Andrés Camilo Muñoz Peña, Michael L. Ariza-Gómez, José Rodríguez, Elizabeth Flórez Yepes

**Affiliations:** † Departamento de Química, Facultad de Ciencias, Universidad Nacional de Colombia − Sede Bogotá, Carrera 30 No., 45-03, Bogotá 111321, Colombia; ‡ Chemistry and Biochemistry Department, 4423 New Mexico State University, Las Cruces, New Mexico 88001, United States; § Centro de Investigaciones en Catálisis, Parque Tecnológico Guatiguará, 28014 Universidad Industrial de Santander, Piedecuesta 681011, Colombia; ∥ Chemistry Division, 8099 Brookhaven National Laboratory, Upton, New York 11973, United States; ⊥ Department of Chemistry, Stony Brook University, Stony Brook, New York 11794, United States; # Instituto de Ciencias Básicas, 27993 Universidad de Medellín, Medellín 050026, Colombia

## Abstract

In this work, we aim to describe the energetics associated with the formation of ammonia from N_2_ interacting with doped hydroxylated rutile TiO_2_(110) surfaces with the vacant O_2c_ site, following the reaction N_2_ + 3H_2_O → 2NH_3_ + 3/2O_2_. The water molecules interact with the surface, creating exposed Ti–OH groups that can transfer hydrogen to the adsorbed N_2_ molecule. Two metal dopants are evaluated: Mo and Ta. For both metals, calculations show a dramatic decrease in the energy of most intermediates during the entire mechanism, leading to more favorable reaction mechanisms. Nonetheless, it is worth noting that when the Ti_6c_ site of the vacant site is doped with either Mo or Ta, there is a stronger effect on the energetics than doping on the exposed Ti_5c_ sites. The effect of increasing the concentration of metal dopants on the vacant site was also investigated. In this case, calculations indicate that a higher percentage of the dopant on the surface results in a more substantial decrease in the energy of most intermediates, suggesting that increasing the dopant content could be beneficial for the catalytic process.

## Graphical abstract

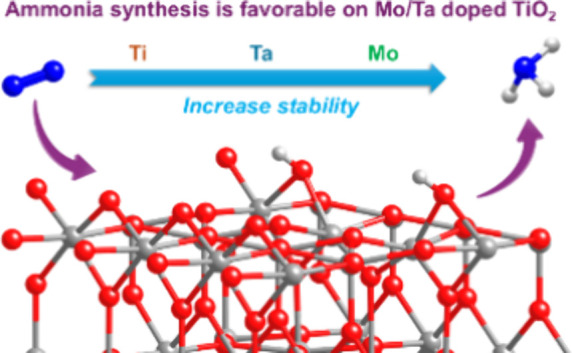

## Introduction

Ammonia is an indispensable chemical with a wide range of applications, including fertilizers, explosives, dyes, plastics, synthetic fibers, and resins.^1^ It is estimated that ammonia is used in over 76% of all nitrogen-based products.^2,3^ Among these applications, fertilizers are the most important, accounting for about 80%, while explosives rank second, indicating a direct impact of ammonia on the agriculture/food and war industries.^2^ Furthermore, in recent years, ammonia has attracted attention as a hydrogen carrier as well.^1^

Given these advantages, ammonia also has a main drawback: its current synthesis. Indeed, almost 95% of the total ammonia production is made through the Haber–Bosch process.^4^ It is efficient, yielding around 97% (Δ*H* ^0^_298_ = −45.9, Δ*G* ^0^_298_ = −16.4 kJ mol^–1^).^1^ However, it is also highly environmentally harmful. In this process, gaseous N_2_ and H_2_ are contacted over an iron-based catalyst at high temperatures (400–600 °C) and pressures (20–40 MPa) with recycling.^1^ These harsh conditions are necessary because gaseous H_2_ is obtained by steam reforming from natural gas and coal, which is a highly endothermic process. As a result, the Haber–Bosch process is responsible for the consumption of 3–5% of the natural gas production and consumes about 1% of the global annual energy generation, with concomitant generation of 1–2% of global CO_2_ emissions.^4–8^ All these environmental concerns demonstrated the urgent need to find alternative approaches for ammonia synthesis that operate at milder conditions of temperatures and pressures and are powered by clean, renewable energy sources.^6^

In recent years, several strategies have been explored, such as homogeneous and heterogeneous catalysis, biomimetic synthesis, and (electro)­photocatalytic approaches. The latter approach has received special attention in recent years since it relies on readily abundant and cheap feedstocks such as air, water, and light and can be done in ambient conditions.^7^ Among various photocatalytic materials, titanium dioxide (TiO_2_) has received most of the attention since the pioneering work by Schrauzer and Guth.^9^ In their work, the authors probed the photolysis of water on incompletely outgassed TiO_2_ powder to H_2_ and O_2_ and the formation of NH_3_ by the reaction of N_2_ with formed H_2_. Additionally, it was demonstrated that doping the catalyst with iron improves the photocatalytic activity.^9^ A few years later, Schrauzer and co-workers also demonstrated that desert sands from a variety of locations around the globe also catalyzed the photoreduction of N_2_ to NH_3_ on exposure to sunlight.^10^ Moreover, the catalytic activity was found to depend on the phase of the crystal, with samples with a high content of rutile being the most active ones.^7^ After these initial contributions on the topic, several works on N_2_ reduction to NH_3_ over TiO_2_ materials appeared in the literature.^11–17^ In a continuous effort to make the process more environmentally friendly, N_2_ reduction has been studied at ambient conditions, using water as a reducing reagent. For instance, Hirakawa and co-workers synthesized NH_3_ from N_2_ and water at atmospheric pressure using commercial samples of TiO_2_ with large amounts of oxygen defects.^18^ A mechanism for the N_2_ reduction and NH_3_ formation was proposed based on diffuse reflectance infrared Fourier transform spectroscopy (DRIFT). It suggests that the active sites are Ti^3+^ ions at the vacancies, which act as adsorption sites for N_2_ and trapping sites for the photogenerated electrons. Thus, N_2_ is successively hydrogenated by surface hydroxyls of the Ti–OH moiety (generated during water splitting) until NH_3_ is generated.

It is well-known that nitrogen fixation on TiO_2_ samples also produces oxidation products such as NO and nitrates.^19^ Both processes, nitrogen reduction and oxidation, involve water and N_2_ splitting. While in the former process, N_2_ is reduced by photogenerated H^+^ and electrons in the conduction band (from water oxidation), in the second process, the photogenerated H^+^ oxidizes N_2_ to NO with water while O_2_ is reduced to H_2_O by the photogenerated electrons. Additionally, it has been suggested^20^ that band alignment for nitrogen oxidation is more favorable than for nitrogen reduction on rutile TiO_2_, which was validated by calculations.^20,21^ Although not totally in agreement with experiments that show nitrogen reduction products, one possible explanation in favor of the observation of NH_3_ is the reduction of NO after nitrogen oxidation, which is also a feasible process. The O_2_ was established to totally suppress NH_3_ formation in the experiments from Hirakawa and co-workers by bubbling air instead of pure N_2_.^18^ While these experimental and computational pieces of evidence demonstrate a critical competition between the oxidation and reduction pathways of N_2_ on TiO_2_, in this work, we are interested in the NH_3_ formation exclusively, and therefore, the exploration of critical reaction pathways is based on the sole formation of NH_3_ using a metal-doped surface with an oxygen vacancy defect.

To molecularly understand the mechanism of N_2_ reduction by water, Thiel and co-workers^22^ used computational chemistry (DFT+D3) to ascertain the detailed reaction pathway for the reaction:

$$N_{2}+3H_{2}O\to 2{\mathrm{NH}}_{3}+3/2O_{2}$$ 1

A 4 × 2 surface slab of rutile(100) with an O vacancy defect was used to simulate the catalyst. According to their calculations, the N_2_ adsorption, N–N bond cleavage, and reduction to NH_3_ are efficiently promoted by the Ti^3+^ ions at the defect sites, and the H_2_O photolysis provides enough energy to overcome the energy barriers (between 0.4 and 1.3 eV). Another important aspect of N_2_ reduction over TiO_2_ materials is the role of metal doping. It has been demonstrated that metals such as Fe, Co, Mo, and Ni enhanced the photocatalytic activity of TiO_2_.^9^ At the computational level, Medford and co-workers analyzed the effects of transition metal dopants in rutile TiO_2_(110) on the direct N_2_ reduction with H_2_ to produce NH_3_.^20,23^ Among all studied metals, Mo, Rh, and Re show better performance compared to pristine TiO_2_, with Mo being the most promising metal.^23^ However, to our knowledge, the effects of metal dopants have not been explored in the ambient reduction of N_2_ with water, and more importantly, the effect of metal dopant concentration has not been addressed.

In this work, we will analyze the energetics of NH_3_ formation via N_2_ reduction with water (eq 1) using models of a doped hydroxylated rutile TiO_2_(110) catalyst with oxygen vacancies as defects. We focus on Mo and Ta as metal dopants. Since the formation of the oxygen vacancy defect induces the formation of Ti^3+^ sites, Mo and Ta dopants also show the same oxidation state. These oxidation states are not unusual for these metals. For instance, molecular complexes of Mo^3+^ have proved to be active in the cleavage of N_2_.^24–26^ On the other hand, heterogeneous catalysts with Ta^3+^ sites have also been involved in the cleavage of N_2_ bond with H_2_.^27–29^

## Methodology and Surface Models

All geometry optimization calculations were carried out at the Perdew–Burke–Ernzerhof (PBE)^30^ level of the Density Functional Theory using VASP.^31–34^ The projector augmented wave (PAW) method was used to describe the inner electrons except for the *d* electrons in all metals, which were treated explicitly.^35^ van der Waals dispersion corrections were employed with the D3 approximation, including Becke–Johnson damping.^36^ The Kohn–Sham orbitals were described using plane waves with a cutoff energy of 450 eV, a value that proved to be suitable for TiO_2_ as reported in the literature.^37^ The presence of dipole moments on the surfaces was also accounted for during calculations by using the dipole correction to the total energy in the *z* direction. To obtain the optimized geometries, the criteria for energy convergence and gradients were set to 10^–6^ eV and 0.01 eV/Å, respectively. A Γ-centered *k-*point mesh of 1 × 1 × 1 was used. In order to verify the dependence of calculated energies on the size of the *k-*point mesh, we also run calculations using a 3 × 3 × 1 mesh for selected steps of the studied mechanism. It is well established that Hubbard (*U*) corrections are necessary to properly describe *d* electrons of transition metals with DFT. However, we do not include *U* parameters in these calculations due to the difficulty of selecting appropriate *U* values for Ti, Mo, and Ta. All calculations in this work were non-spin-polarized, since the energies and geometries from spin-polarized vs non-spin-polarized were very similar (vide infra). Energies reported in the article are based on pure electronic energies at 0 K, as delivered by VASP. However, for selected pathways, we also compute Gibbs energies at 298 K to verify the entropy and temperature dependence.

Several surface models of rutile TiO_2_ were created by using the periodic slab approach. We start with a pristine rutile(110) surface (no defects or dopants), created from the experimental bulk structure reported in the Materials Project database.^38^ From this structure, a *k*-point convergence study was first conducted, where cell parameters are converged as a function of the *k*-point mesh. A (8 8 13) *k*-point grid was selected since it provided bulk parameters (*a* = *b* = 4.650 Å, *c* = 2.966 Å) below 0.12% compared to the experimental values, indicating that the methodology used here is suitable for describing the lattice structure of rutile.^39^ Then, a 4 × 2 surface model slab exposing the (110) facet was created using VESTA.^40^ The resulting supercell has 48 Ti atoms and 96 O atoms, accommodated in three O–Ti–O trilayers. A 20 Å vacuum was added to the surfaces in the *z* direction to accommodate the incoming reactants, products, and intermediates as well as to avoid interaction with adjacent images. During geometry optimizations, the bottom three layers were kept fixed at bulk geometry, while the upper three were allowed to relax together with the adsorbed species. Figure 1a shows the pristine rutile (110) surface, in which the most important exposed atoms are highlighted. From this surface, a hydroxylated surface with an oxygen vacant defect was created by removing one O_2c_ and adding hydrogen atoms to the oxygen atoms adjacent to the vacant site (see Figure 1b). Next, we created four different surfaces with dopant metals Mo and Ta, replacing one and two surface Ti ions next to the vacant site by the corresponding metals (see Figure 1c for the Mo-doped surface as an example), generating doped surfaces with 2.1 and 4.2% doping metals, respectively. The substitution energies for the two dopants with respect to the undoped surface were calculated by using eq 2:

$$\Delta E_{\mathrm{subs}}=E_{\mathrm{surf},M-\mathrm{doped}}-E_{\mathrm{surf},\mathrm{undoped}}+(E_{\mathrm{Ti}}-E_{M})$$ 2

where *E*_surf,M‑doped_ is the energy of the doped system (either with one or two metal dopant atoms), *E*_surf,undoped_ is the energy of the pristine, undoped surface, *E*_Ti_ is the energy per atom of the bulk titanium metal, and *E*_M_ is the energy per atom of the bulk tantalum and molybdenum metals. Eq 2 approximates the substitution energies as if the metal dopant (Ta or Mo) is brought into the pristine surface from the bulk metallic form, and the displaced Ti atom is incorporated into the titanium bulk. Calculations carried out here indicate that the substitution energies are 4.65 and 0.90 eV for Mo and Ta dopants, respectively, for the substitution of one Ti atom. In the case of double-doped systems, substitution energies are 8.85 and 2.09 eV, respectively, for Mo and Ta. The first values are similar to those already reported in the computational literature.^23^ Although the value of 8.85 eV for the double-doped system with Mo is extraordinarily high to overcome in an experimental synthesis experiment, we decided to keep this surface in the present work for the sake of systematicity. We also must point out here that a variety of synthesis methods to incorporate metals into the TiO_2_ structure operate under nonequilibrium conditions, effectively overcoming thermodynamic limitations by driving the system far from equilibrium to obtain stable or metastable structures in which metallic dopants can be incorporated into the TiO_2_ lattice. Therefore, high calculated energies do not necessarily imply that experimental synthesis is unattainable.

**Figure 1:**
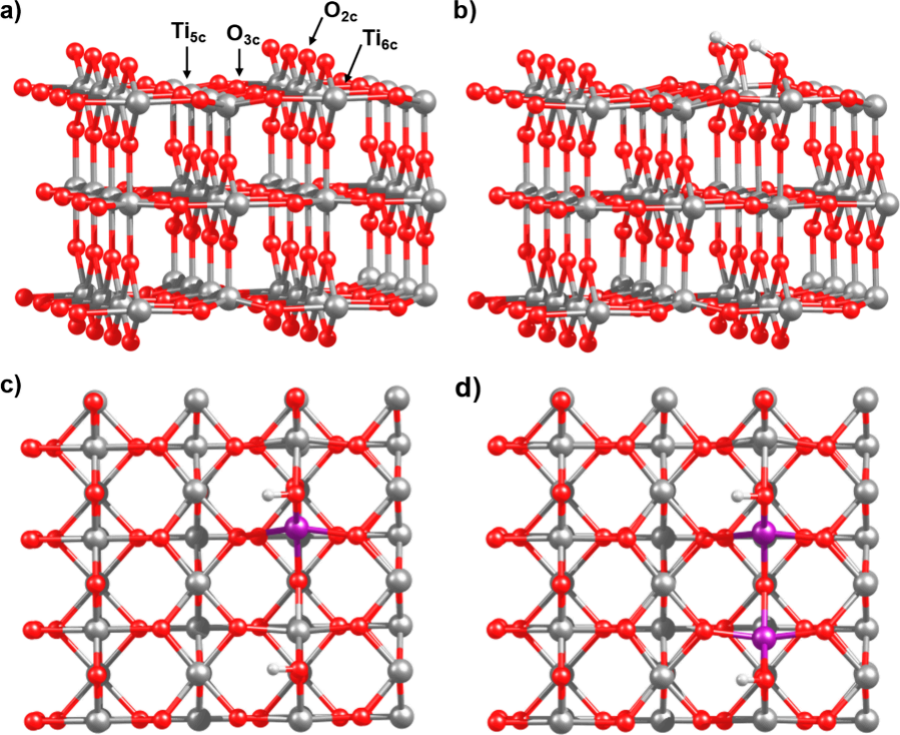
Optimized surfaces of (a) the pristine rutile TiO_2_(110) surface with the most relevant exposed atoms highlighted; (b) the hydroxylated rutile TiO_2_(110) surface with an O_2c_ vacancy; (c) the hydroxylated rutile TiO_2_(110) surface with an O_2c_ vacancy doped with one atom; (d) the hydroxylated rutile TiO_2_(110) surface with an O_2c_ vacancy doped with two atoms.

Several experimental^41^ and theoretical studies^42^ showed that surface and subsurface metal (V, Sb, Sn, Zr, and Hf) doping has roughly the same probability of occurring. Moreover, a recent study using multiple techniques like powder X-ray diffraction (PXRD), field-emission scanning electron microscopy (FE-SEM), X-ray photoelectron spectroscopy, and diffuse reflectance (DR) UV–vis spectroscopy, among others, showed that increasing the Mo doping % tends to enrich the surface of TiO_2_ nanoparticles rather than the bulk.^43^ Therefore, in this work, we decided to explore surface doping at the oxygen vacancy defect, as it enables direct binding to the N_2_ molecule.

The mechanism investigated herein will be based on the associative distal pathway, which was previously identified as the most energetically favored pathway.^22^ In this mechanism, the N_2_ molecule is first absorbed on the active site and then successive hydrogenations on the distal (outer) N atom allow N–N bond breaking and the release of the NH_3_ molecules, as shown in Scheme 1. Since it is well-known that the dissociative pathway requires very high energy input to cleave the N–N bond and that hydrogenation on the directly adsorbed N atom to the surface (associative alternating mechanism) is restricted by steric reasons,^22^ these mechanisms are not considered in this work. However, it must be pointed out that some other alternative mechanisms will be explored thoroughly.

**Scheme 1:**
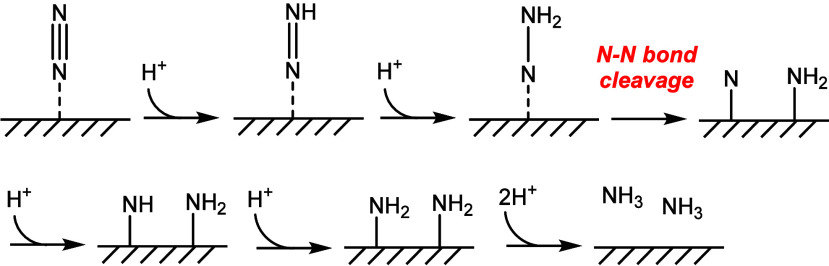
Associative Distal Mechanism for NH_3_ Formation from N_2_ and a Hydroxylated Surface

Finally, we must emphasize here that other factors such as solvent, pH dependence, and potential effects are not accounted for in this work. This is because we are more interested in energy trends across different doping patterns than in actual adsorption energies. Nevertheless, all of these factors will be explored in subsequent work for the most energetically feasible reaction pathways.

## Results

The results of this work will be organized as follows: first, we will present the results of the N_2_ adsorption and reduction at the M_5c_ site (M = Ti, Mo, and Ta) of the hydroxylated rutile TiO_2_(110) surface and on the Ti_5c_ site when the vacant site is doped with Mo and Ta. Then, the effect of the doping of one Ti_6c_ site of the vacant O_2c_ site on the associative distal mechanism for NH_3_ formation is presented. Finally, the last section shows a discussion of the effect of increasing the doping content on the vacant site on the reaction energetics.

The nomenclature used for identifying the reaction intermediates consists of consecutive numbers with a subscript indicating the metal (Ti, Mo, and Ta); for instance, **1**_**Ti**_. In the case of doping two Ti^3+^ sites of the vacant site, the subscript will indicate the number 2 before the metal, that is, **1**_**2Ti**_. For the initial N_2_ adsorption on different sites (M_5c_ and M_6c_), the reference energy is always the bare surface + N_2_.

### N_2_ Adsorption, Hydrogenation, and Activation over Hydroxylated M_5c_ Sites with O_2c_ Vacancy Defects

The first surface hydroxylation takes place by the reaction of a defective TiO_2_ rutile surface with H_2_O prior to N_2_ adsorption and activation, which creates two M–OH groups next to the O vacant site and releases a half equivalent of O_2_. Starting from this surface, we explored different mechanisms for N_2_ activation and hydrogenation at the exposed M_5c_ sites of the hydroxylated TiO_2_ rutile(110) surface. Figure 2 shows the energy profile for the proposed mechanism, while Table S1 and Figures S1–S3 of the Electronic Supporting Information (ESI) show geometrical parameters and optimized structures. In both the M_5c_ and M_6c_ sites, the N_2_ coordinates to the metal center in an end-on mode, consistent with previous studies.^22,44,45^ Regardless of the metal dopant, the adsorbed states have a lower energy than the separate reactants, suggesting that N_2_ adsorption is a favorable process. Moreover, the energies of intermediates **2**_**M**_ are very similar among different metals (−0.29/–0.34 eV), indicating that the metal dopants have little effect on the N_2_ adsorption process. In these intermediates, the N–N bond distances are 1.11 Å, which is the same distance as in isolated N_2,_ and thus no N_2_ activation is observed upon adsorption. The M_5c_–*N bond lengths range from 2.49 to 2.60 Å. Hydrogenation at the distal N atom in **2**_**M**_ generates **3**_**M**_. For M = Mo, Ta, the *NNH species is successfully obtained during surface optimization (see inset panel in Figures 2, S2, and S3), but for M = Ti, such a species could not be located. Instead, the adsorbed N_2_ remains as *NN species (see Figure S1) and the proton originally in the distal N atom migrates to another O_2c_ atom of the surface.

**Figure 2:**
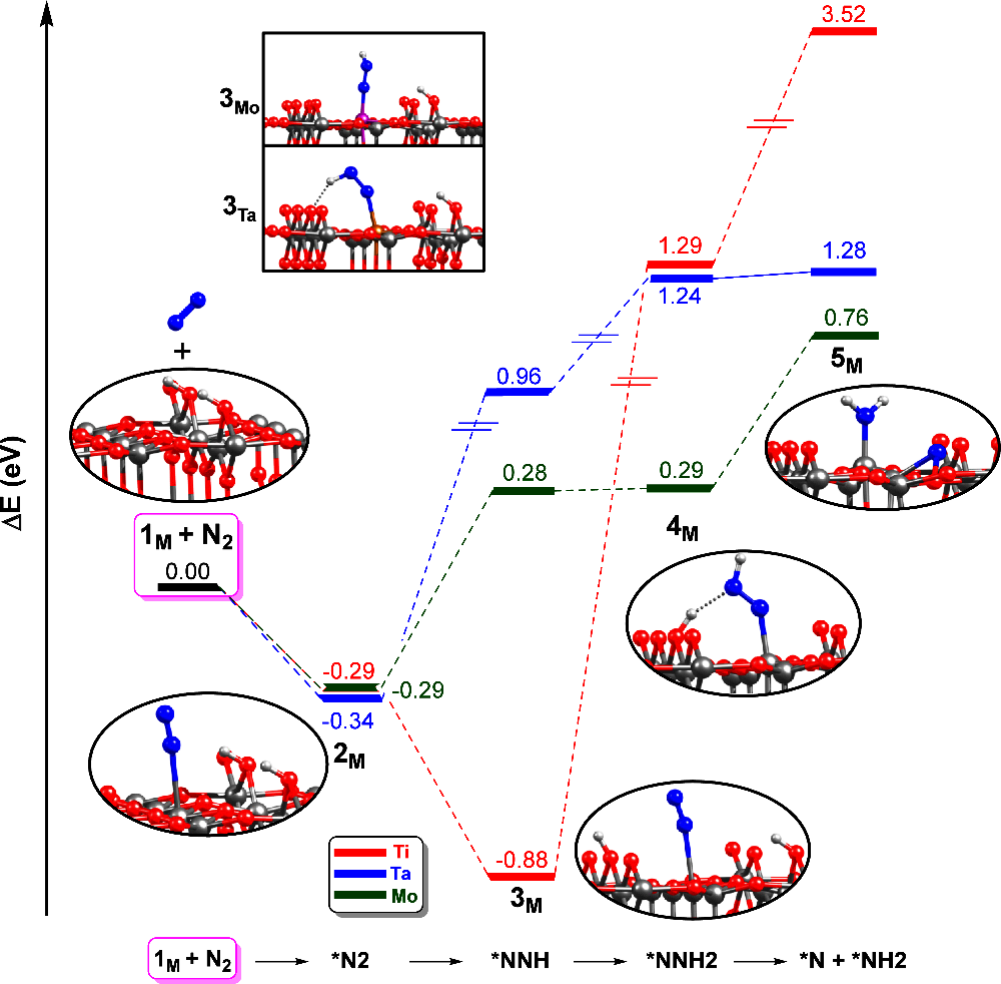
Energy profiles (in eV) of the N_2_ adsorption, hydrogenation, and activation over M_5c_ sites (M = Ti, Mo, and Ta) of the hydroxylated rutile TiO_2_(110) surface with an O_2c_ vacancy defect. The origin of energies is the bare surface (**1**_**M**_) and N_2_.

As can be seen for **4**_**M**_, hydrogenation of the *NN fragment at the distal N atom is a highly unfavorable process. While for Ti and Ta, the energies are 1.24/1.29 eV, for Mo, **4**_**M**_ is only 0.29 eV above the initial reactants. The calculations show that the Ti_5c_ site of the undoped surface is unable to cleave the N_2_ bond, given the high energy of **5**_**Ti**_ (3.52 eV). Doping with Mo and Ta has a beneficial effect, with energy lowering of 2.76 and 2.24 eV, respectively. Nevertheless, the energies of **5**_**Mo**_ and **5**_**Ta**_ are still very high compared to the reactants to be considered a feasible process. We also investigated the reactivity of Ti_5c_ sites when the Ti_6c_ sites of the vacant site of the O_2c_ are doped with Mo and Ta. The corresponding energy profile is shown in Figure S4 of the ESI along with geometrical parameters (Table S2) and optimized structures for Mo and Ta (Figures S5 and S6). As can be seen, the *N–N bond breaking is still prohibitive, and thus these surfaces are also unable to activate N_2_ at the Ti_5c_ site. In general, it seems that although Mo/Ta doping helps stabilize some intermediates during N_2_ hydrogenation and activation, the M_5c_ sites are unable to successfully activate N_2_ regardless of the position of the dopant metal. This prompted us not to investigate further N_2_ hydrogenation reactions at this site.

### N_2_ Adsorption, Hydrogenation, and Activation over Hydroxylated M_6c_ Sites with O_2c_ Vacancy from First H_2_O Photolysis

When N_2_ interacts with the M_6c_ of the O_2c_ vacant site, the same pathway as discussed for the M_5c_ site applies. Figure 3 shows the energy profile, and Table S3 in ESI shows the most relevant geometrical parameters. The optimized structures are shown in Figures S7–S9 of ESI. In all cases, the N_2_ coordinates to the M sites of the vacant site in the end-on mode. N_2_ adsorption is favorable for the undoped and doped surfaces (**6**_**M**_), with Ti and Ta having similar energies (−0.28/–0.31 eV), and Mo showing the most stable intermediate, **6**_**Mo**_ (−0.87 eV). This trend nicely correlates with the geometric parameters of the adsorbed species: while **6**_**Ti**_ and **6**_**Ta**_ show long M–*N and Ti–*N bond distances (2.93 and 2.84/2.96 Å, respectively), **6**_**Mo**_ shows Mo–*N bond distances of 1.98/2.70 Å. Moreover, the *N–N bond distance is slightly longer in **6**_**Mo**_ compared to **6**_**Ti**_ **/6**_**Ta**_ (1.11 vs 1.15 Å), suggesting slight N_2_ activation. At this point, it is worth comparing the results obtained here with those available in the recent literature that use TiO_2_ surfaces with vacancies. Two main studies have been published on the subject. The first study from Medford and co-workers^23^ studied the trends in activity of N_2_ reduction to NH_3_ on transition metal-doped surfaces with no hydroxylation. For the N_2_ adsorption on the vacancy, they found energy values of 0.13, −0.10, and −0.27 eV for Ti-, Ta-, and Mo-doped surfaces. Although these values are a bit different from ours, the trend in adsorption energies is the same, indicating some reproducibility of our data. The second study from Thiel and co-workers^22^ focuses on the same reaction as we study here, that is, the reduction of N_2_ with hydroxylated surfaces with vacancy defects in the undoped TiO_2_ surface. The N_2_ adsorption energy is −0.61 eV, which differs from our data by about 0.26 eV, probably because they used a different methodological setup for the final energies. However, the most important difference lies in the structure of the N_2_ adsorbed state. While the two Ti–N distances are 2.93 Å in the present study, they reported values of ∼2.4 Å, that is, about 0.5 Å shorter than our values. This is surprising because the setup for geometrical optimization of the surface slabs is the same for both studies, theirs and ours. All our efforts to obtain a similar structure for N_2_ adsorption were unsuccessful, and all calculations led to the same structure **6**_**Ti**_, as shown in Figures S7. To date, we do not know the origin of this discrepancy.

**Figure 3:**
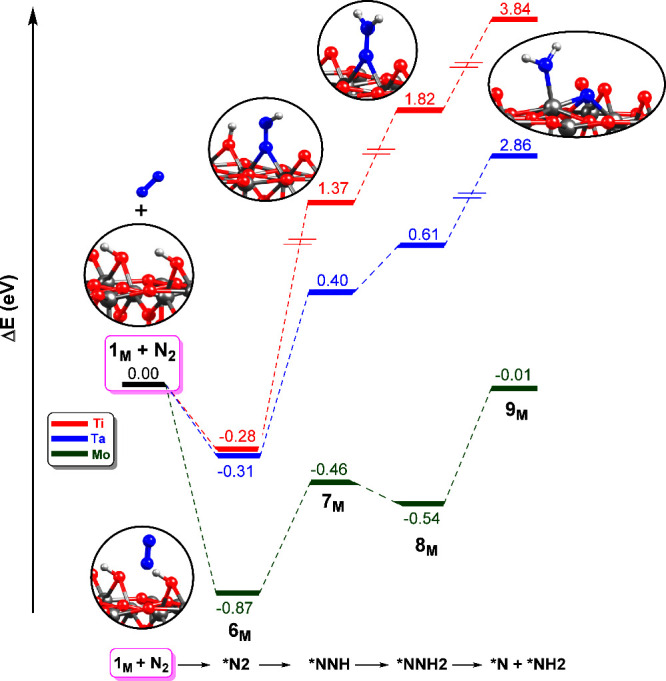
Energy profiles (in eV) of the N_2_ adsorption, hydrogenation, and activation over M_6c_ sites (M = Ti, Mo, Ta) of the hydroxylated rutile TiO_2_(110) surface with an O_2c_ vacancy defect, using a 1 × 1 × 1 *k* point mesh. The origin of energies is the bare surface (**1**_**M**_) and N_2_.

Focusing on M = Ti, in **6**_**Ti**_, the successive hydrogen transfers from the two vicinal hydroxyls generate **7**_**Ti**_ (*NNH) and **8**_**Ti**_ (*NNH2), with energies of 1.37 and 1.82 eV. In these intermediates, the *N–N bond distance is vaguely longer (1.22/1.23 Å) than in **6**_**Ti**_, suggesting partial N_2_ activation, which is accompanied by a shortening of the two Ti_6c_–*N bonds (2.14/2.25 and 2.22 Å, for **7**_**Ti**_ and **8**_**Ti**_, respectively, see Table S3). From **8**_**Ti**_, the N–NH2 bond cleavage gives **9**_**Ti**_, in which the NH_2_ fragment is transferred to a neighboring Ti_5c_ site, and the *N atom remains bonded to the defect vacant site. This step, however, features a very high energy intermediate (3.84 eV) and an energetic bottleneck of more than 4 eV (with respect to **6**_**Ti**_, the lowest energy intermediate), indicating that bond breaking of N_2_ does not occur from **8**_**Ti**_.

It is clear from Figure 3 that doping the vacant site with Mo and Ta is beneficial for the N_2_ hydrogenation and even N_2_ bond breaking, since these intermediates are stabilized by at least 1 eV, and in the case of Mo, the energies of **7**_**Mo**_ and **8**_**Mo**_ remain below the reference energy. In **7**_**Mo**_ and **7**_**Ta**_, the N–N bond length is similar to the value for **7**_**Ti**_ (1.23 Å), but in **8**_**Mo**_ and **8**_**Ta**_, the N–N bond length is slightly longer. While for **7**_**Ta**_ and **8**_**Ta**_ the Ta–*N and Ti–*N bond lengths are very similar to the values of the undoped system, in **7**_**Mo**_ and **8**_**Mo**_, the Mo–*N bond length is shorter than the Ti–*N bond length, indicating a stronger interaction. This could be related to the higher stability of these intermediates compared to the undoped and Ta-doped surface. From **8**_**Ta**_ and **8**_**Mo**_, the N–N bond cleavage can also occur in a way similar to that of the undoped system, leading to **9**_**Ta**_ and **9**_**Mo**_. Again, this step is highly endoenergetic for both metals, although the extent is greater for Ta than for Mo. Indeed, while for Ta the breaking of the N_2_ bond costs about 2.2 eV (with respect to **8**_**Ta**_), for Mo, this step costs only 0.5 eV. Overall, the energetic bottleneck for Ta is more than 3 eV, and for Mo, it is less than 1 eV (with respect to **6**_**M**_, the lowest energy intermediate). These results suggest that Ta is also unable to activate the N_2_ bond from **8**_**Ta,**_ and only Mo doping can lead to an active catalyst for N_2_ activation. Nevertheless, a barrier of 0.86 eV for Mo is still a bit high for a photocatalytic process, and other mechanisms for N_2_ activation can be investigated. The energies calculated here for the undoped surface (M = Ti) show some qualitative coincidence with the work of Thiel and co-workers,^22^ especially the high energy of intermediate **8**_**Ti**_.

Three methodological aspects deserve attention at this point: *k*-point mesh, entropy and temperature effects, and spin polarization effects on the calculated energies. In order to test the reliability of our results with the 1 × 1 × 1 *k-*point mesh, we also calculate the energies of all intermediates involved in the first N_2_ adsorption, hydrogenation to *NH2 over the M_6c_ sites, and N–N activation on the O_2c_ vacancy defect (Figure 3). The energy profiles and geometrical parameters are shown in Figure S10 and Table S3 in the ESI, respectively. As observed, increasing the *k*-point mesh does not affect the energy trends shown in Figure 3. Moreover, the change in the *k*-point set lowered the energies of all intermediates by only 0.16 eV on average. Table S3 also shows that the main geometrical parameters are practically unaffected by this new *k*-point set. These results point to the fact that the calculated energy trends are independent of the *k*-point set, and the results shown with the 1 × 1 × 1 mesh are reliable. We also compute the Gibbs energies at 298 K for the pathways shown in Figure 3. The results are shown in Figure S11 of the ESI. The net result of including ZPE and entropy effects is an increase of ∼0.5 eV (average) on the energies compared to the electronic energies at 0 K. In this case, adsorption of N_2_ on the pristine (undoped) and Ta-doped materials becomes slightly endergonic (0.16 eV for Ti and 0.10 eV for Ta). Meanwhile, it remains favorable for Mo (0.40 eV below the reference energy), indicating that doping with Mo is beneficial for the reaction. More importantly, as the effect of ZPE and entropy is homogeneous along the pathways, all energy trends are conserved. For instance, for the Mo-doped pathway (the green one in Figure S11), the energetic barrier between the lowest and highest energy intermediates is 0.86 eV (**6**_**Mo**_ and **9**_**Mo**_), the same value as computed with electronic energies (Figure 3). These results suggest that using electronic energies can be reliable in discussing the reaction energetics along the different metal dopants. Finally, test calculation using spin polarization on the mechanisms shown in Figure 3 demonstrates that relative energies are slightly affected (see Table S4 in ESI), and very similar results are found for spin-polarized and non-spin-polarized calculations. In view of this, all calculations are done without spin polarization.

### N_2_ Activation and Hydrogenation over Hydroxylated M_6c_ Sites with O_2c_ Vacancy from Second H_2_O Photolysis

Calculations in the previous subsection showed that N_2_ activation in **8**_**M**_ is a very energetically demanding process of at least 1 eV for Mo (see Figure 3). Thus, we now focus on alternative mechanisms. One possibility might be that the reaction takes place on a hydroxylated surface rather than on a nonhydroxylated surface like **8**_**M**_. The experimental work of Hirakawa et al. demonstrated NH_3_ formation on defective TiO_2_ sites under an aqueous environment (200 mL water) and N_2_ bubbling,^18^ indicating that water is readily available for subsequent surface rehydroxylation. We thus created a new model from **8**_**M**_ with two M–OH groups, namely **10**_**M**_, as shown in Figures 4 for **10**_**Ti**_ and in Figures S13 and S14 of ESI for **10**_**Mo**_ and **10**_**Ta**_, respectively. The structure of the *NNH2 fragment in **10**_**M**_ is similar for the three considered surfaces, with slightly larger *N–N bond distances and shorter M–*N bond lengths with respect to those of **8**_**M**_. From **10**_**M**_, we investigated two possible reaction pathways for N_2_ bond cleavage and subsequent hydrogenation. The first mechanism involves direct N_2_ cleavage from *NNH2 in **10**_**M**_ (Figure 4, left), while the second one considers first *NNH2 hydrogenation from one of the vicinal −OH, leading to *NNH3 and *N, and then N_2_ bond breaking (Figure 4, right). The corresponding geometric parameters can be found in Tables S5 and S6 and the optimized structures in Figures S12–S17 in ESI.

**Figure 4:**
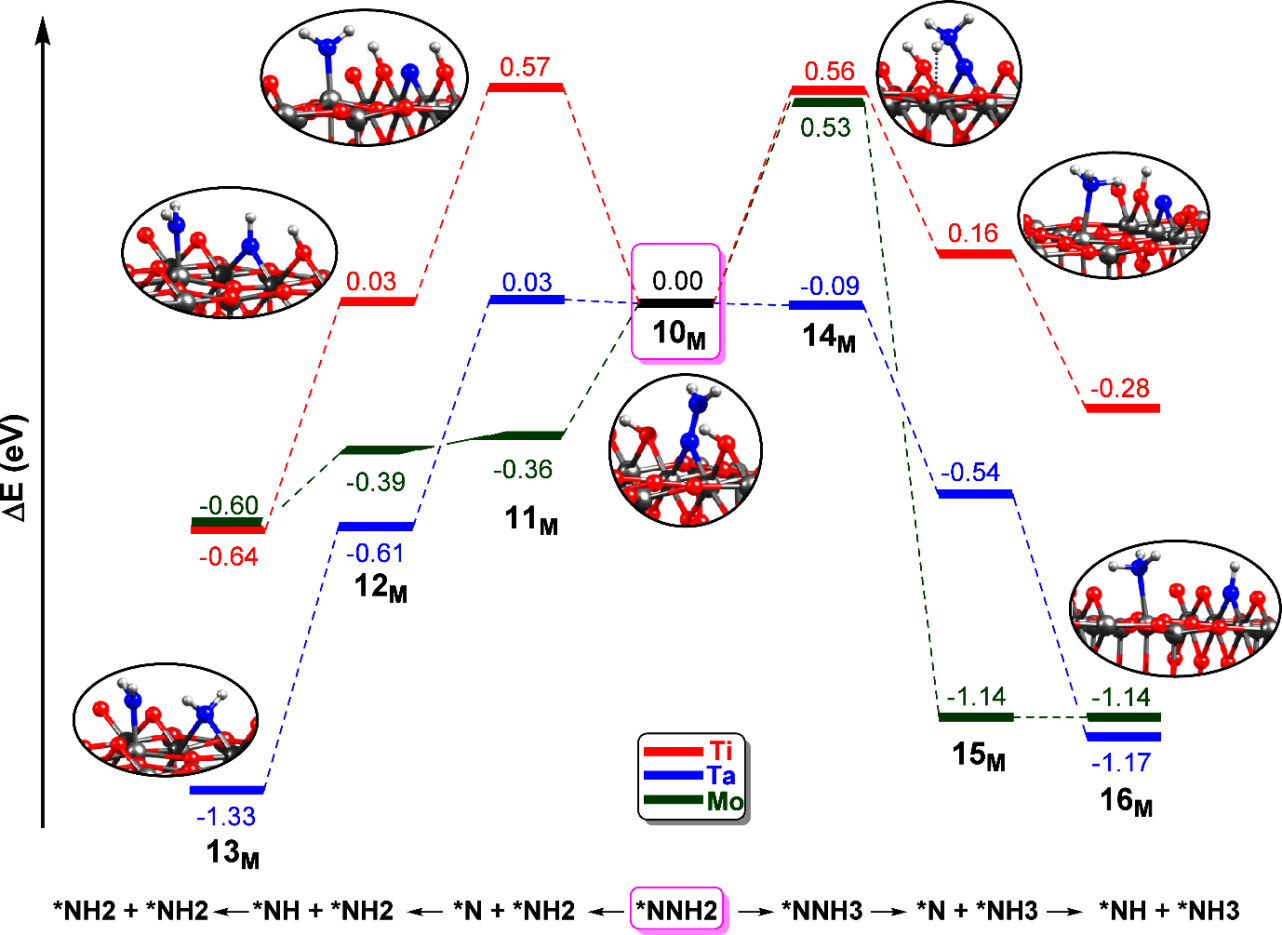
Energy profiles (in eV) of the N_2_ activation and hydrogenation over M_6c_ sites (M = Ti, Mo, Ta) of the hydroxylated rutile TiO_2_(110) surface with an O_2c_ vacancy defect, starting from **10**_**M**_ (*NNH2 species), following two mechanisms: direct N–N bond cleavage to *NH2 and *N (left) and first hydrogenation to *NNH3 and then N–N bond cleavage to *NH3 and *N (right). The origin of the energies is intermediate **10**_**M**_.

Focusing on the first pathway, the breaking of the N_2_ bond from *NNH2 species is highly dependent on the metal dopant. In this step, the NH_2_ fragment is transferred to the exposed Ti_5c_ of the surface, leading to **11**_**M**_. The geometries of the new *NH2 adsorbed species are similar for all three metals, with Ti5c–*N distances of 1.89 Å. However, the atomic *N species adsorbed on the O_2c_ vacancy shows a more asymmetric distribution of the two M–*N bond lengths for Mo (1.71/2.27 Å) compared to Ti and Ta, where both lengths are more evenly distributed (1.83 and 1.90/1.82 Å, respectively), see Table S5. From the energetic point of view, the stability of the adsorbed species follows the trend Mo > Ta > Ti, indicating that doping with Mo and Ta was favored for N_2_ activation. Thus, while **11**_**Ti**_ shows an energy of 0.57 eV, **11**_**Ta**_ and **11**_**Mo**_ show energies of 0.03 and −0.39 eV. From **11**_**M**_ successive proton transfer from the Ti–OH groups to adsorbed *N leads to **12**_**M**_ and **13**_**M**_. For all three metals, this mechanism is downhill, although the stability of these intermediates is dependent on the metal identity. Indeed, while Ti and Mo give hydrogenated species of similar stability (−0.64 and −0.60 eV, respectively), Ta gives the most stable adsorbed species, **13**_**Ta**_, at −1.33 eV with respect to **10**_**Ta**_.

On the other hand, by looking at the second mechanism (right side of Figure 4 and Table S6), similar observations to the previous ones can be made. The first step in this pathway is a proton transfer from the Ti–OH groups to the distal N atom of the *NNH2 moiety, leading to **14**_**M**_. It must be pointed out, however, that the corresponding *NNH3 species could only be obtained for Ti since Mo and Ta cannot stabilize such a species. For these metals, the hydrogen that is transferred to the NH2 fragment remains bonded to the bridging O_2c_ (see Figures S16 and S17). Several attempts to optimize the *NNH3 species for these two metals were unsuccessful. For Ti and Mo, the energies of **14**_**M**_ are similar (0.56 and 0.53 eV, respectively), while for Ta, this species is only slightly lower in energy than **10**_**Ta**_ (−0.09 eV). In **14**_**M**_, the N_2_ bond cleavage takes place, leading to **15**_**M**_, where a *NH3 is transferred to a Ti_5c,_ and one *N remains adsorbed on the vacancy. In **15**_**M**_, the Ti_5c_–*N bond lengths range from 2.19 (Ti) to 2.23 Å (Mo), while the bond distances M–*N range from 1.72 to 2.24 Å; see Table S6. The reaction **14**_**M**_ → **15**_**M**_ is exothermic in all cases, but the energy stabilization of **15**_**M**_ is different. **15**_**Mo**_ gives the most stable species (−1.14 eV) and **15**_**Ti**_ the least stable at 0.16 eV, with **15**_**Ta**_ having an intermediate value of −0.54 eV. The next proton transfer from Ti–OH to the *N species gives **16**_**M**_. For Ti and Ta, there is a stabilization of these species compared to that of **15**_**M**_, and for Mo, it remains at the same energy. Thus, similar to the first mechanism considered here, hydrogenation of *N adsorbed species is also a downhill process, with Mo and Ta giving similar energetics.

It is interesting now to compare the two proposed mechanisms for the formation of hydrogenated species **13**_**M**_ and **16**_**M**_. For the undoped system (Ti), both pathways show similar energetics, since intermediates **11**_**Ti**_ and **14**_**Ti**_, the energetic bottleneck of the reactions, show the same energy, although **13**_**Ti**_ is more stable than **16**_**Ti**_. A similar statement can be drawn for Ta, for which there is no energetic bottleneck, and both mechanisms occur below **10**_**Ta**_ in energy (except for **11**_**Ta**_, which is only 0.03 eV above **10**_**Ta**_). As for Ti, **13**_**Ta**_ is more stable than **16**_**Ta**_. For Mo, there is a difference between the two mechanisms, since the sequence **10**_**Mo**_ ··· → ··· **13**_**Mo**_ occurs below the energy of reactant **10**_**Mo**_, while intermediate **14**_**Mo**_ lies at 0.53 eV above reactant. Thus, calculations suggest that for the Mo-doped catalyst, the direct N_2_ cleavage from **10**_**Mo**_ is the preferred pathway. Finally, comparison of the reaction energies for the N_2_ cleavage processes during first and second water photolysis, that is, comparison of reaction **8**_**M**_ → **9**_**M**_ (Figure 3) with **10**_**M**_ → **11**_**M**_ (Figure 4) suggests that surface hydroxylation assists the N_2_ bond cleavage favorably. While for the nonhydroxylated surface, this reaction is endoenergetic by 0.5 eV for Mo and 2.2 eV for Ta (with respect to **8**_**M**_), for the hydroxylated surface, the reaction energies become favorable for Mo and are isoenergetic for Ta. This effect has been attributed to the distribution of the two generated electrons after hydroxylation with H_2_O, in which one electron is distributed at the vacant site, and the other is transferred to the π* orbital of the N–N bond.^22^

### Hydrogenation of *NH2 Species over Hydroxylated M_6c_ Sites with O_2c_ Vacancy from Third H_2_O Photolysis

The final stage of the studied mechanism of NH_3_ formation from N_2_ is the hydrogenation of adsorbed *N species **13**_**M**_ and **16**_**M**_. This step can happen only on a hydroxylated rutile TiO_2_(110) surface, and thus, we created new models for these surfaces, which include two Ti–OH groups in the vicinity of the vacant site. The energy profile is shown in Figure 5, Table S7 presents the most relevant geometrical parameters, and Figures S18–S20 display the optimized structures. The new surface model, **17**_**M**_, is structurally similar to **13**_**M**_ (compare the geometrical parameters in Tables S5 and S7), indicating that hydroxylation does not greatly affect the geometry of these structures. From **17**_**M**_, two possible proton transfers generate two different intermediates, namely, **18**_**M**_ and **19**_**M**_. In the **17**_**M**_ → **18**_**M**_ step, the proton transfer occurs from the *NH2 fragment located on the O_2c_ vacancy to the *NH2 on the Ti_5c_ site, leading to a *NH3 species on this site (this is equivalent to forming the hydroxylated species **18**_**M**_ from **16**_**M**_). This transfer elongates the Ti_5c_–*N bond from 1.92/1.94 Å in **17**_**M**_ to 2.22/2.24 Å in **18**_**M**_, while the two M–*N and Ti–*N bonds at the vacancy become shorter. The formation of **19**_**M**_ passes by proton transfer from one Ti–OH group to the *NH2 fragment adsorbed on the vacancy. In this case, only the two M–*N and Ti–*N bonds become larger than their values in **17**_**M**_, and the Ti_5c_–*N bonds remain unaffected. From the energetic point of view, species **19**_**M**_ is always lower than **18**_**M**_, regardless of the metal involved, indicating that formation of a *NH3 species on the O_2c_ vacancy is preferred over formation of a *NH3 species on the Ti_5c_ site. The degree of surface stability depends on the metal identity. Ti shows the highest energy difference, ranging from 0.25 eV in **18**_**Ti**_ to −0.31 eV in **19**_**Ti**_, while for Ta and Mo, the differences are smaller. In the latter case, species **18**_**Mo**_ and **19**_**Mo**_ are very similar in energy.

**Figure 5:**
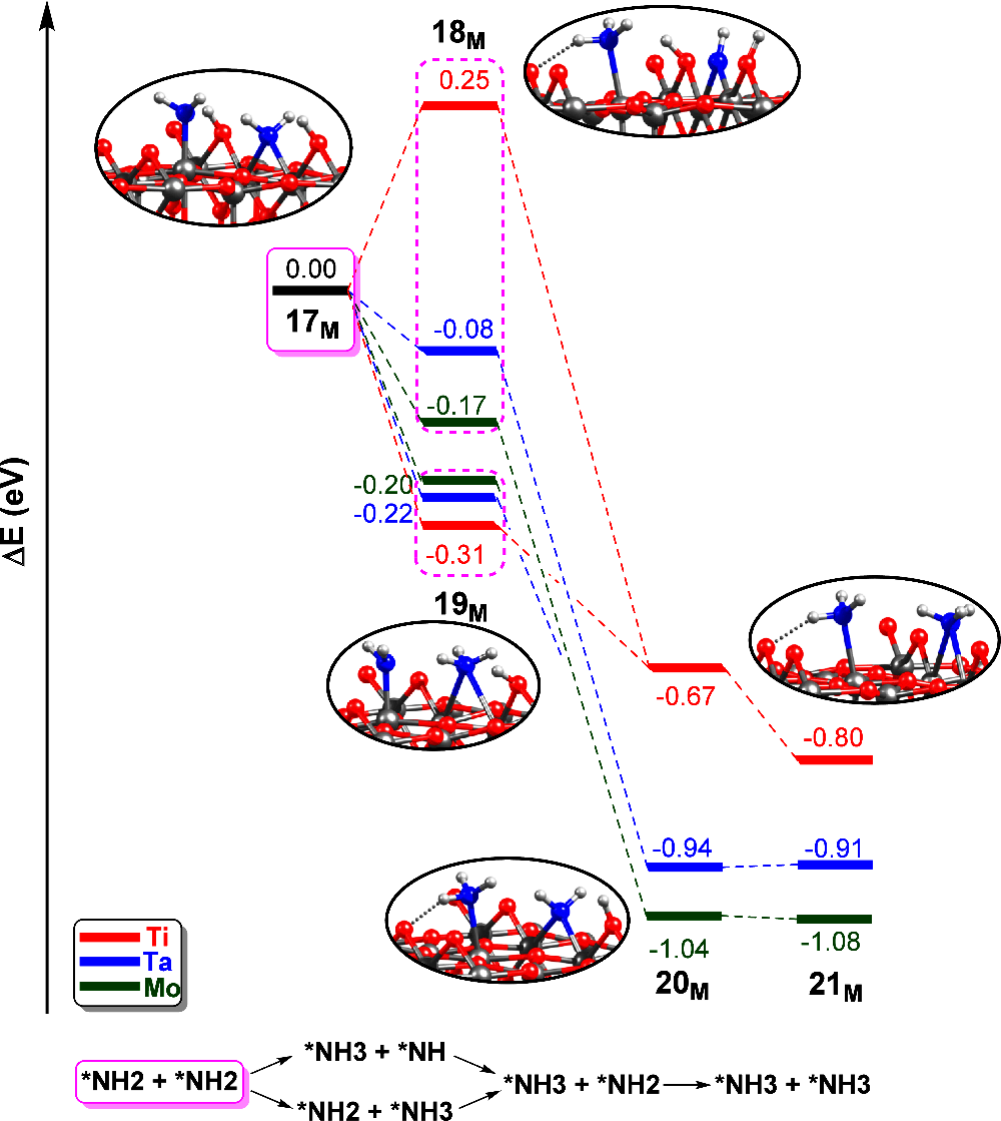
Energy profiles (in eV) of the *NH_2_ hydrogenation to NH_3_ over M_6c_ sites (M = Ti, Mo, Ta) of the hydroxylated rutile TiO_2_(110) surface with an O_2c_ vacancy defect. The origin of the energies is intermediate **17**_**M**_.

Both intermediates **18**_**M**_ and **19**_**M**_ lead to the same species **20**_**M**_, which presents *NH3 adsorbed on Ti_5c_ and *NH2 adsorbed on the vacant site. The step **17**_**M**_ → **20**_**M**_ takes place by a proton relay mechanism in which a proton is first transferred to the *NH2 fragment at the vacancy and then to the *NH2 at the Ti_5c_. Finally, the last transfer of a proton from the Ti–OH group to *NH2 leads to two *NH3 species adsorbed on Ti_5c_ and on the vacancy, **21**_**M**_. The energies of both **20**_**M**_ and **21**_**M**_ are well below that of **17**_**M**_, suggesting that hydrogenation of *N species is energetically favored. As in previous discussions, the undoped catalyst shows the least stable intermediates, while doped systems with Mo and Ta lead to the stabilization of all intermediates. In this case, both doped surfaces show very similar energy values (−0.94/0.91 eV for Ta and −1.04/–1.08 eV for Mo). From **21**_**M**_, the two NH_3_ molecules can be released to the gas phase.

Overall, the calculations have shown a dramatic effect of metal doping, and both metals, Mo and Ta, are demonstrated to be promoters of the N_2_ bond breaking and reduction to NH_3_ over hydroxylated surfaces. The analysis indicates that direct doping at the Ti_5c_ site results in catalysts that are less efficient compared to those doped at the Ti_6c_ sites of the O_2c_ vacancy. Therefore, the last sites are explored and studied more thoroughly here. In particular, Mo has been shown to stabilize most of the intermediates compared to the undoped surface, in agreement with previous calculations,^23^ indicating that Mo doping will have a direct impact on the mechanism of NH_3_ formation from N_2_ on a hydroxylated surface. Ta also showed an increase in the catalytic activity, and this is most obvious for the reactions of the second and third hydroxylation processes. During the first stage of N_2_ adsorption and hydrogen transfer, the energies of Ta intermediates are higher in energy than those of the Mo-doped catalyst but still lower than those of the undoped system.

In the next section, we explore the effect of increasing the amount of metal doping at the Ti_6c_ sites of the O_2c_ vacancy. To this end, we created new models of the surfaces with two metal dopants on the vacant site for both Mo and Ta; see Figure 1d.

### Effect of Metal Doping Concentration on the Energetics of NH_3_ Formation

All mechanisms described in previous sections rely on the fact that only one six-coordinated Ti^3+^ on the vacant site is replaced by a metal dopant, Mo and Ta, corresponding to ∼2% of metal doping. In this section, we investigate the effects of increasing the amount of doping to ∼4% by replacing two Ti^3+^ ions with the O vacancy with two metals (see Figure 1d). In this case, we also compute the mechanism of NH_3_ formation based on three stages, as presented above for the hydroxylated surfaces with an O_2c_ defect. In general, most of the intermediates are similar in nature to those already discussed, although some geometric variations emerge as a consequence of having two doping atoms in the vacancy instead of one. Here, we will discuss the most relevant aspects of the effect of increasing the metal dopant content in the original material TiO_2_.

Figure 6 shows the energetic profiles of the first stage of the reaction, namely, the initial N_2_ adsorption on the O_2c_ vacancy and subsequent hydrogen transfer to the distal N atom. Table S8 shows the relevant geometric parameters, and Figures S21 and S22 show the optimized geometries of all intermediates for Mo and Ta doping. During this stage, it is observed that doping with two metal atoms helps to stabilize all intermediates compared to doping with only one (compare Figures 3 and 6). The extent of surface species stabilization ranges from −3.08 eV (**9**_**2Ta**_) to −0.23 eV (**7**_**2Mo**_), which results from comparing the same intermediate with one and two doping metal atoms. Moreover, in contrast to the intermediates with one Ta dopant, all the new intermediates show energies below the initial reactants, suggesting that the energetics with Ta doping is more sensitive to the content of metal doping than those of Mo. It is interesting to highlight that, for these new catalysts with higher doping content, the N_2_ bond breaking is feasible even for the nonhydroxylated surface, which is in contrast to the case of the catalysts with only one metal dopant atom (Figure 3). For Mo doping, the energy of **9**_**2Mo**_ is −1.19 eV, and the energetic bottleneck is imposed by **6**_**2Mo**_ (−1.46 eV), leading to 0.27 eV of difference between the two intermediates. This difference is much smaller than that of the analogous mechanisms (Figure 3, 0.86 eV). Similarly, for Ta, the energetic bottleneck is reduced to 0.42 eV (energy difference between **8**_**2Ta**_ and **9**_**2Ta**_), a very low value compared to the analogous mechanism with one metal dopant (Figure 3, 3.17 eV). From the geometric point of view, most intermediates preserve the trends discussed previously. However, significant changes in some bond distances are observed, especially in the M1–*N and M2–*N lengths (see Table S8). It is worth noting that for intermediate **6**_**2Ta**_, the N_2_ coordinates to the vacant site in a side-on form, which is mostly preserved in **7**_**2Ta**_ (see Figure S22). These are the only cases of side-on coordination of N_2_ found in this work.

**Figure 6:**
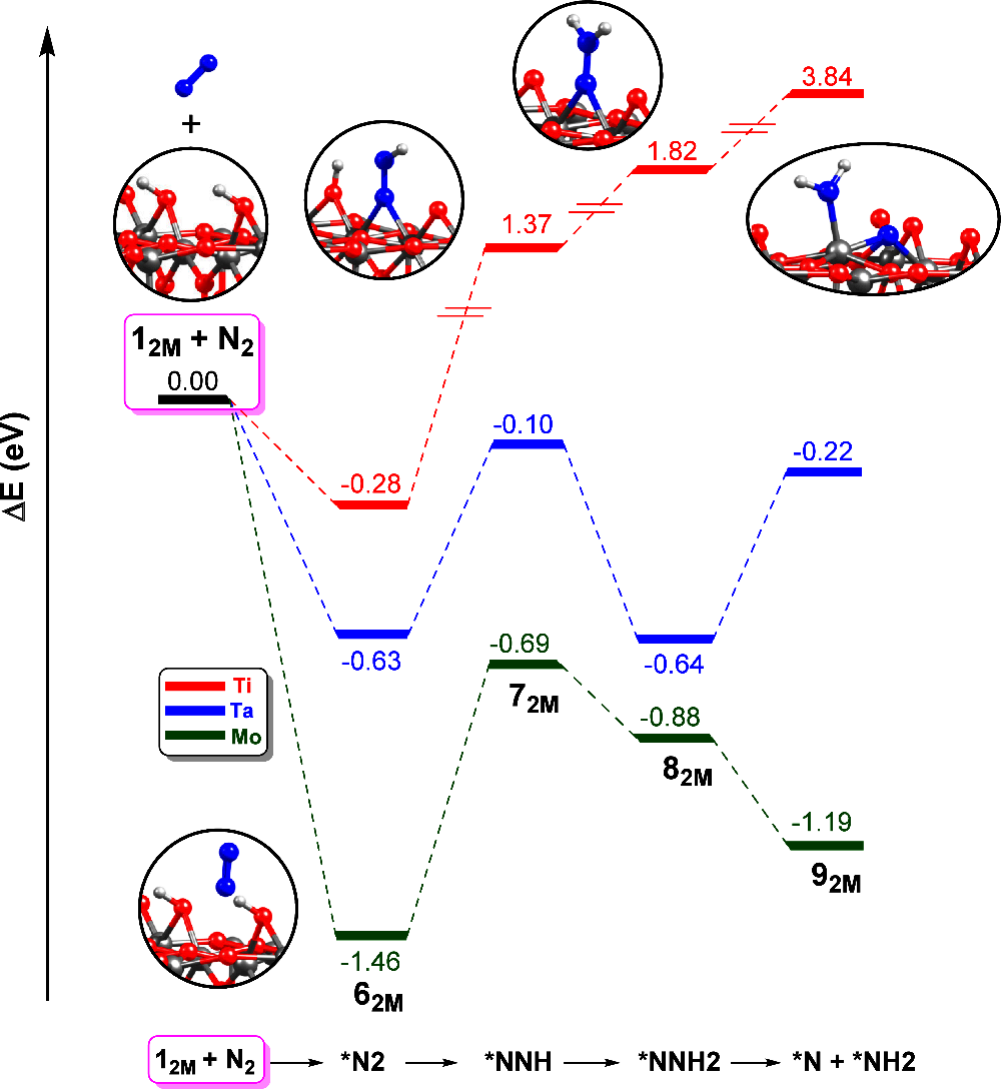
Energy profiles (in eV) of the N_2_ adsorption and activation over M_6c_ sites (M = Ti, Mo, Ta) of the hydroxylated rutile TiO_2_(110) surface with an O_2c_ vacancy defect with two doping atoms in the vacancy. The origin of the energies is the bare surface (**1**_**2M**_) and N_2_.

After the initial N_2_ adsorption and reduction, the next stage includes the other possible N_2_ bond cleavage mechanisms concomitant with subsequent reductions, in a mechanism analogous to the one shown in Figure 4. The new energy profiles are shown in Figure S23 of ESI, the relevant geometrical parameters can be found in Tables S9, S10, and Figures S24–S27 show the optimized geometries for Mo and Ta doping. Again, surface rehydroxylation produces **10**_**2M**_, from which hydrogenation and N–N bond-breaking reactions occur. Analogous mechanisms, as previously discussed, were analyzed (Figure 4), that is, direct N_2_ cleavage to *NH2 and *N, and first hydrogenation to *NNH3, and then N–N bond cleavage to *NH3 and *N. As in the previous stage, all intermediates **11**_**2M**_–**16**_**2M**_ show energies lower than those associated with the catalyst with one metal dopant (compare Figures 4 and S23), with energy stabilization going from −1.31 (**14**_**2Ta**_) to −0.07 eV (**11**_**2Mo**_). Interestingly, for the surface doped with Mo, the energy of **11**_**2Mo**_, the intermediate formed by cleavage of the N_2_ bond in **10**_**2Mo**_, is −0.43 eV. This value is higher than that of **9**_**2Mo**_ (Figure 6, −1.19 eV), suggesting that N_2_ bond breaking is more favorable in a nonhydroxylated surface. For Ta, the hydroxylated surface gives the most favorable pathway for N_2_ bond breaking. Thus, calculations again demonstrate that increasing the metal doping content at the vacant site is beneficial for the mechanisms explored here.

Finally, the last stage comprises the hydrogenation of the *NH2 species formed in the last step. Again, we use a hydroxylated surface, **17**_**2M**_. The results can be found in Figures S28–S30 and Table S11. In sharp contrast to the previous stages, in this case, doping with two metal atoms does not always lead to intermediates lower in energy than those of one metal dopant. In fact, in the case of Mo doping, all intermediates increase their energy, with **19**_**2Mo**_ having the most dramatic effect (i.e., an increase of 1.51 eV). For Ta, most intermediates decrease their energies with respect to the one Ta doping, except **19**_**2Ta**_ (which increases by 0.26 eV). These findings just indicate that in this case, the formation of **19**_**2Mo**_ is unfavorable compared to **18**_**2Mo**_, and the mechanism would prefer the formation of *NH3 and *NH species and the relay mechanism for *NH3 formation (**17**_**2Mo**_ → **18**_**2Mo**_ → **19**_**2Mo**_), which is unfavorable for Mo by 0.40 eV. The same reaction sequence applies for the Ta-doped surface, but in this case, the intermediates **20**_**2Ta**_ and **21**_**2Ta**_ are well below the reference energy.

## Conclusions

The current need to look for more environmentally friendly solutions for obtaining ammonia has led researchers to investigate alternatives to the classic iron catalysts used in the Haber–Bosch process. Among all the tested catalysts, TiO_2_ has been well investigated due to its photocatalytic properties. In particular, this catalyst has been used in the reduction of N_2_ via the photolysis of H_2_O. Moreover, metal doping has shown an increase in the reaction yield. However, the real effect of the doping metals has not been addressed in detail. Thus, in this work, we focused on studying the effect of Mo and Ta on the reaction energetics for the N_2_ + 3H_2_O → 2NH_3_ + 3/2O_2_ process using periodic DFT calculations of a surface model of hydroxylated rutile TiO_2_(110) with an O_2c_ vacant site. Our results show that the Ti_6c_ sites at the vacancy are more reactive than the exposed Ti_5c,_ and doping of Ti_6c_ sites with Mo and Ta leads to stabilization of most of the intermediates of the associative distal mechanism. In general, this stabilization is more pronounced for Mo than for Ta. The use of hydroxylated surfaces, which result from the reaction of pristine TiO_2_ with water by photolysis, is beneficial for the reaction.

Finally, we also investigated the effect of increasing the concentration of the metal dopant at the vacancy. In this case, we considered two Mo and Ta atoms, and calculations showed a stronger stabilization of most of the intermediates. These results show that the reaction outcome could be, in principle, modulated by modifying the percentage of metal dopant in the original material. A summary of all reaction mechanisms for the N_2_ reduction to NH_3_ at the M_6c_ sites is shown in Figures 7–9. From these figures, the role of the type and amount of metal dopants is more evident. During the first stage (Figure 7) energy stabilization is observed for all intermediates of the doped surfaces, although the extent of this stabilization depends upon the surface species. It is observed that Mo provides the surface with a more favorable reactivity. During the second surface hydroxylation (Figure 8), we also observed stabilization of most intermediates of the doped surfaces, and in general, such stabilization is greater for the surfaces doped with two metal atoms. During the third stage (third surface hydroxylation, Figure 9), we found intermediates that were stabilized upon doping, but some of them also increased their energy. It is also observed that increasing the Mo doping in this state is not favorable to the reaction mechanism. Thus, it seems that increasing the metal dopant content does not always have positive effects on the reactivity of the TiO_2_ surface.

**Figure 7:**
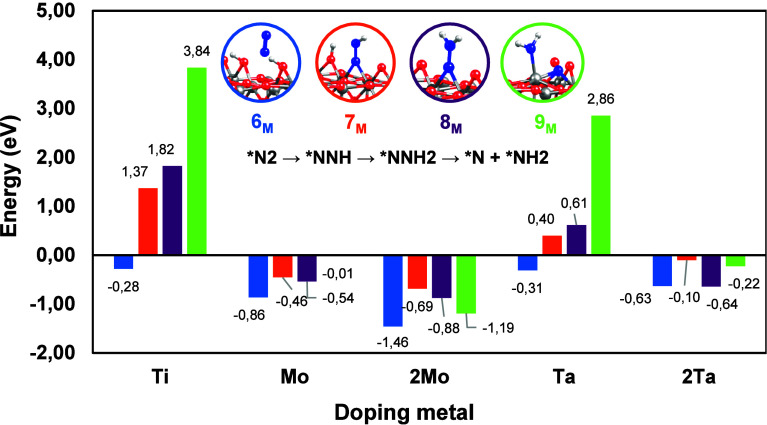
Summary of the reaction mechanism of the N_2_ adsorption, hydrogenation, and activation over M_6c_ sites (M = Ti, Mo, Ta) of the hydroxylated rutile TiO_2_(110) surface with an O_2c_ vacancy defect with one and two doping metal atoms. The colors of the bars correspond to the colors of each structure.

**Figure 8:**
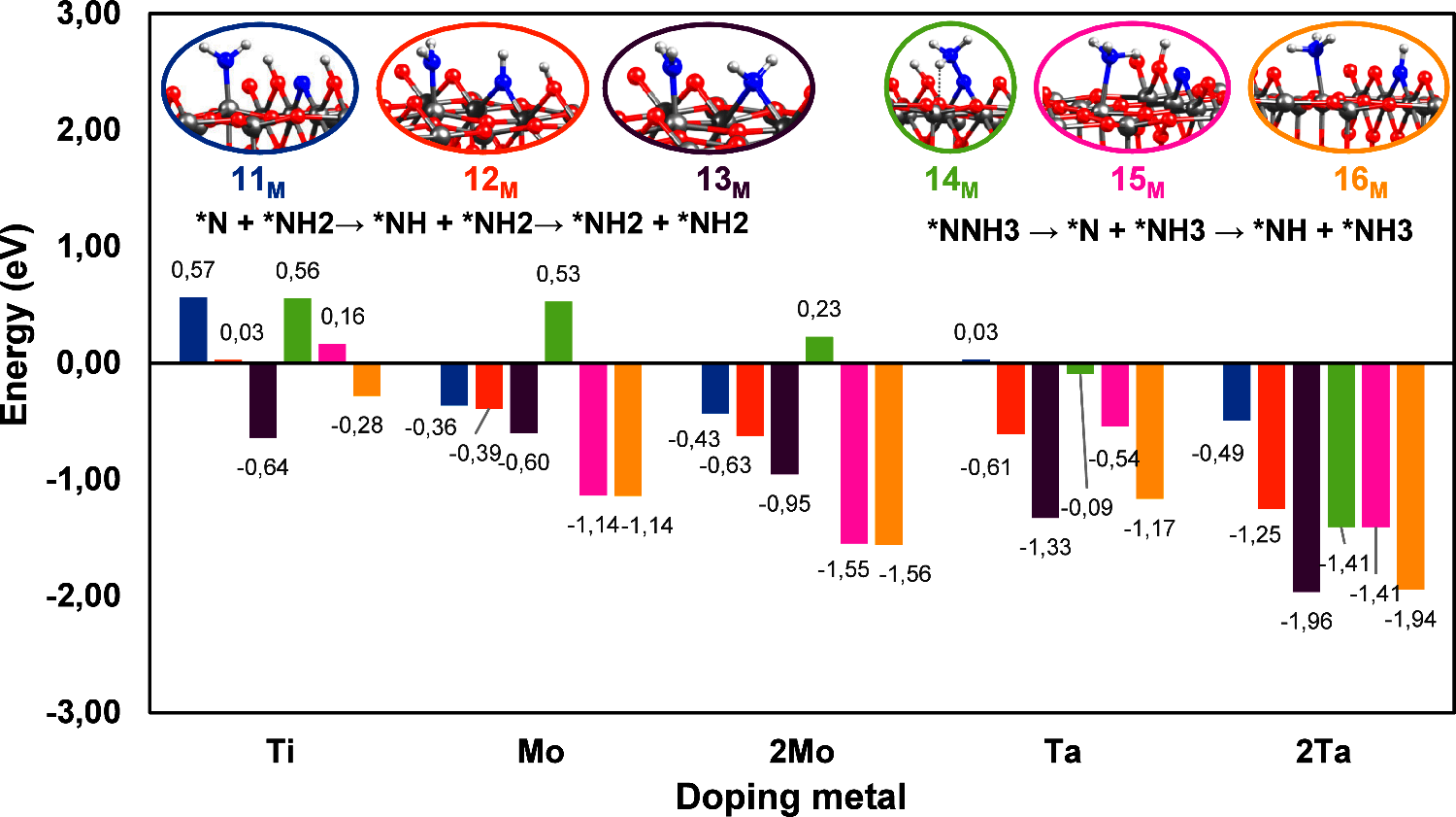
Summary of the reaction mechanisms of the N_2_ activation and hydrogenation over M_6c_ sites (M = Ti, Mo, Ta) of the hydroxylated rutile TiO_2_(110) surface with an O_2c_ vacancy defect. Two pathways were considered: first cleavage of the *N–N bond and then proton transfer (left) and first proton transfer and then cleavage of the N–N bond. The colors of the bars correspond to the colors of each structure.

**Figure 9:**
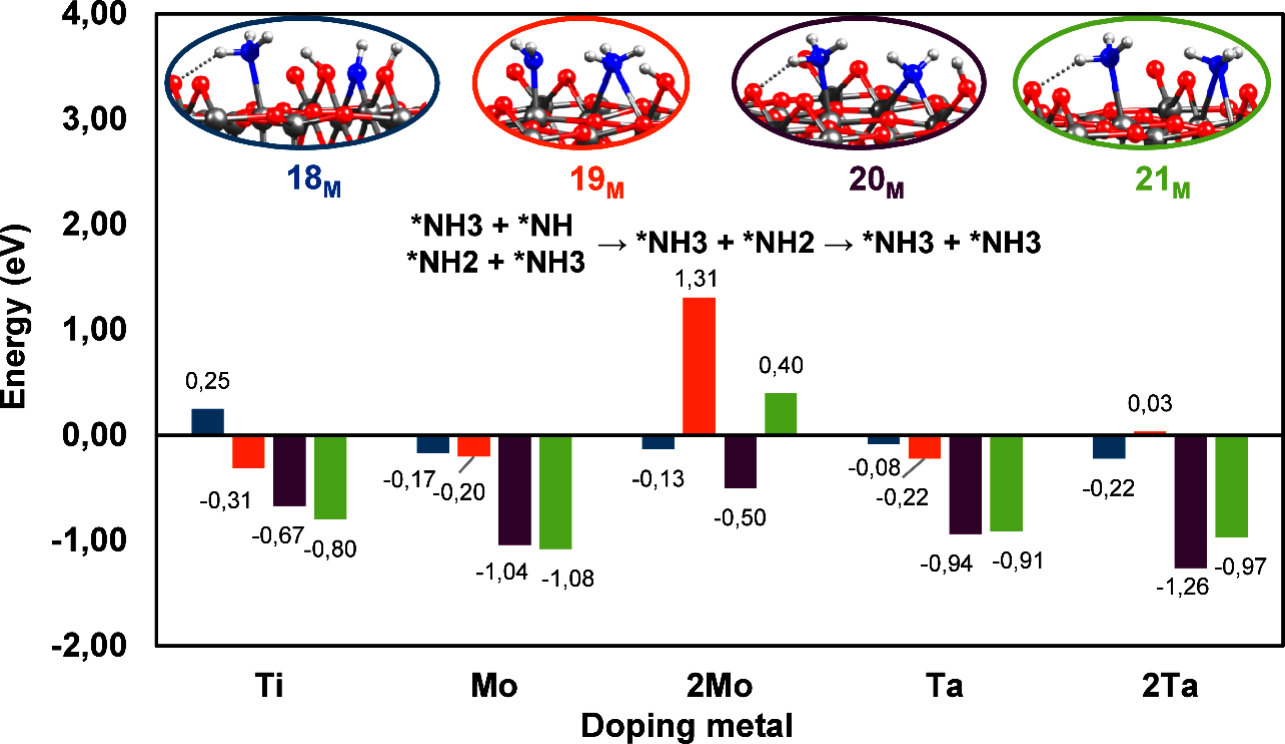
Summary of all reaction mechanisms of the *NH_2_ hydrogenation to NH_3_ over M_6c_ sites (M = Ti, Mo, Ta) of the hydroxylated rutile TiO_2_(110) surface with an O_2c_ vacancy defect. The colors of the bars correspond to the surrounding colors of each structure.

We strongly believe that our current work contributes significantly to the search for more efficient materials for synthesizing ammonia. However, the calculations shown here are just one small step, as several aspects remain unexplored. For instance, the effects of entropy and temperature are only partially accounted for here, and energy barriers are not considered for the study of kinetics. These two aspects can bring light to the feasibility of the kinetics of reactions, and this type of calculation is currently in progress in our groups.

## Supplementary Material



## Notes

Tables with relevant geometrical parameters, optimized structures of all intermediates, energy profiles for N_2_ activation and hydrogenation over Ti_5c_ sites with doped Ti_6c_ of the oxygen vacancy, energy profiles of the N_2_ activation and hydrogenation over Ti_6c_ of the oxygen vacancy using a 3 × 3 × 1 *k*-point mesh, Gibbs energy profiles of the N_2_ activation and hydrogenation over Ti_6c_ of the oxygen vacancy, comparison of spin-polarized vs non-spin-polarized calculations, and energy profiles for the second and third water photolysis stages of the systems with two doping metal atoms (PDF)
