# Supplementary material for: Catalytic Ammonia Synthesis over Pure, Defective, and Metal-Doped Rutile TiO2: A Periodic DFT Study
Source: J Phys Chem C Nanomater Interfaces. 2026 Jan 27;130(6):2148–59. doi: 10.1021/acs.jpcc.5c06031 (PMC12908153; doi:10.1021/acs.jpcc.5c06031)
Supplement: Supplementary file 1 [file jp5c06031_si_001.pdf]

Supporting Information

Catalytic Ammonia Synthesis over Pure, Defective and Metal-Doped Rutile TiO_2: A Periodic DFT Study

Francisco Núñez-Zarur,[1,*] Andrés Camilo Muñoz Peña,[2] Michael L. Ariza-Gómez,[3] José Rodríguez,[4,5] Elizabeth Flórez Yepes[6]

[1]Departamento de Química, Facultad de Ciencias, Universidad Nacional de Colombia – Sede Bogotá, Carrera 30 No., 45-03, 111321 Bogotá, Colombia

[2]Chemistry and Biochemistry Department, New Mexico State University, 88001, Las Cruces, NM, USA

[3]Centro de Investigaciones en Catálisis, Parque Tecnológico Guatiguará, Universidad Industrial de Santander, 681011 Piedecuesta, Colombia.

[4]Chemistry Division, Brookhaven National Laboratory, Upton, New York 11973, United States

[5]Department of Chemistry, Stony Brook University, Stony Brook, New York 11794, United States

[6]Instituto de Ciencias Básicas, Universidad de Medellín, 050026 Medellín, Colombia

Table S1. Relevant distances (in Å) of all intermediates involved in the N_2 adsorption and activation over M_{5c} (M= Ti, Mo, Ta) sites of the of the hydroxylated rutile TiO_2 (110) surface with an O_{2c} vacancy defect. See Figure 2 for energies.

	**Metal**	***N-N**	**M_{5c}-*NN**	**N/O_{2c}···H**	**Ti_{5c}-*NH2**	**Ti-N***
	**Ti**	1.11	2.60			
**2_M**	**Mo**	1.11	2.49			
	**Ta**	1.11	2.58			
	**Ti**	1.11	2.60			
**3_M**	**Mo**	1.22	1.80			
	**Ta**	1.20	2.03	1.74		
	**Ti**	1.22	2.02	1.80		
**4_M**	**Mo**	1.30	1.77	1.80		
	**Ta**	1.31	1.82			
	**Ti**	4.35			1.88	1.93
**5_M**	**Mo**	3.06			1.88	1.67
	**Ta**	1.42			2.26	1.86

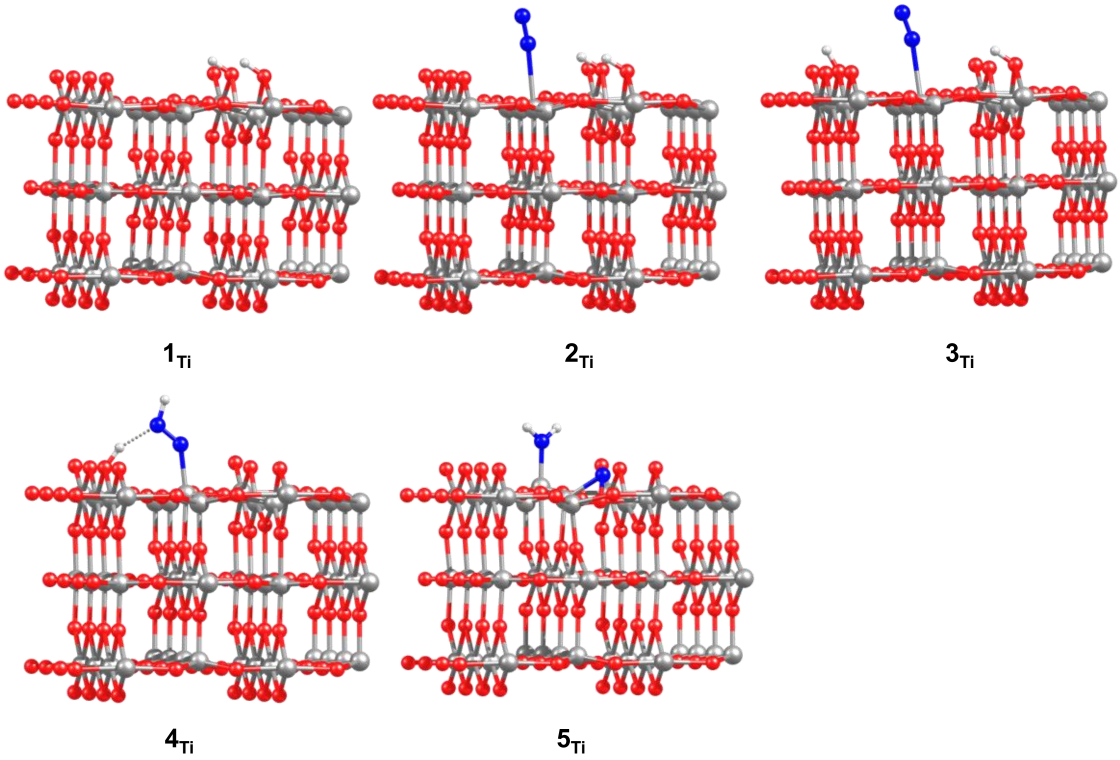

Figure S1. Optimized structures of the intermediates involved in the N_2 adsorption and activation over Ti_{5c} site of the hydroxylated rutile TiO_2 (110) surface with an O_{2c} vacancy defect.

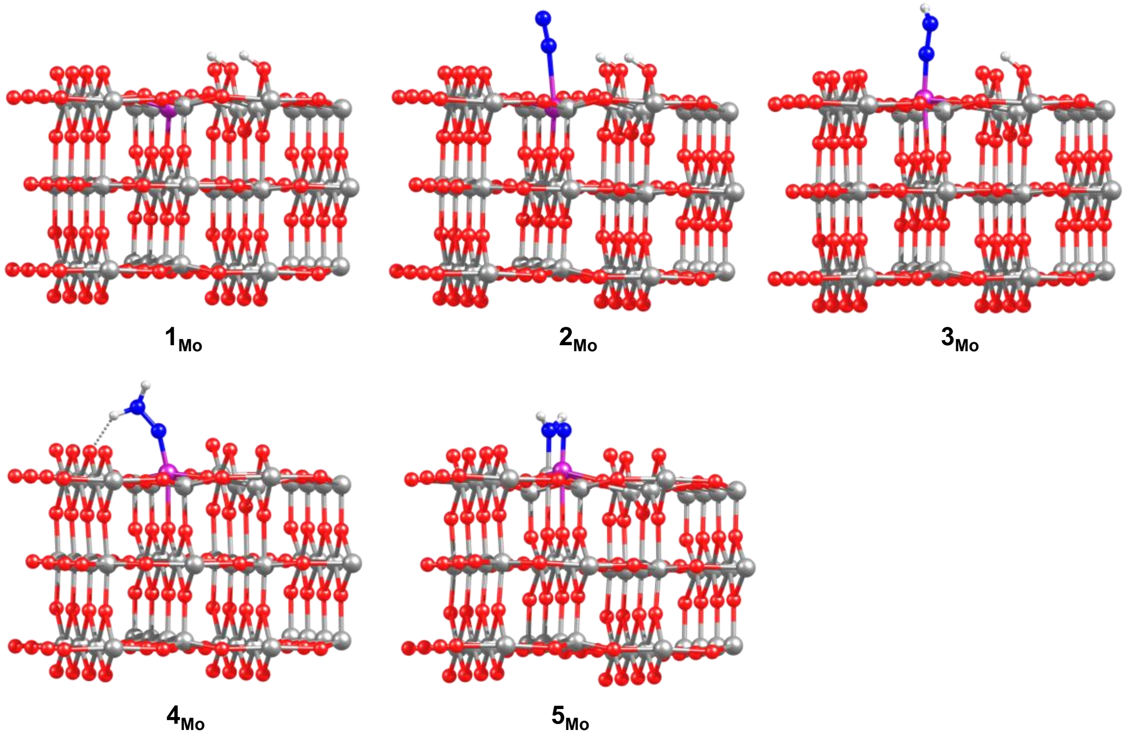

Figure S2. Optimized structures of the intermediates involved in the N_2 adsorption and activation over Mo_{5c} site of the hydroxylated rutile TiO_2 (110) surface with an O_{2c} vacancy defect.

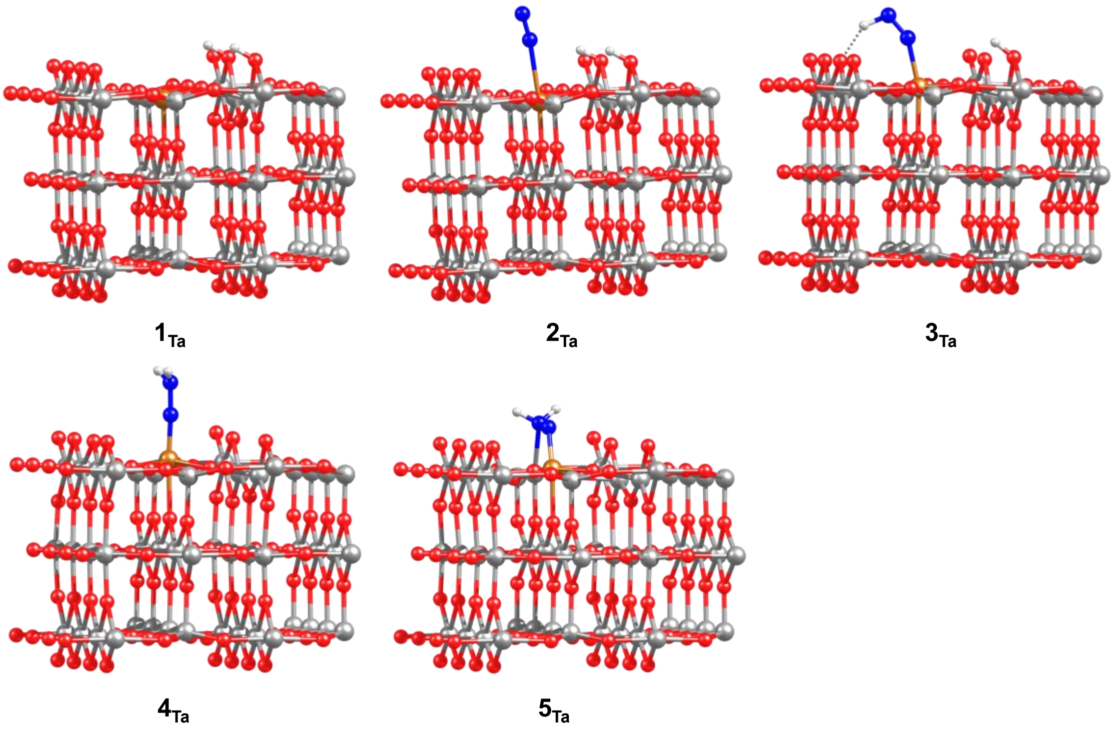

Figure S3 panel labels: $\mathbf{1}_{Ta}$, $\mathbf{2}_{Ta}$, $\mathbf{3}_{Ta}$, $\mathbf{4}_{Ta}$, $\mathbf{5}_{Ta}$

Figure S3. Optimized structures of the intermediates involved in the N_2 adsorption and activation over Ta_{5c} site of the hydroxylated rutile TiO_2 (110) surface with an O_{2c} vacancy defect.

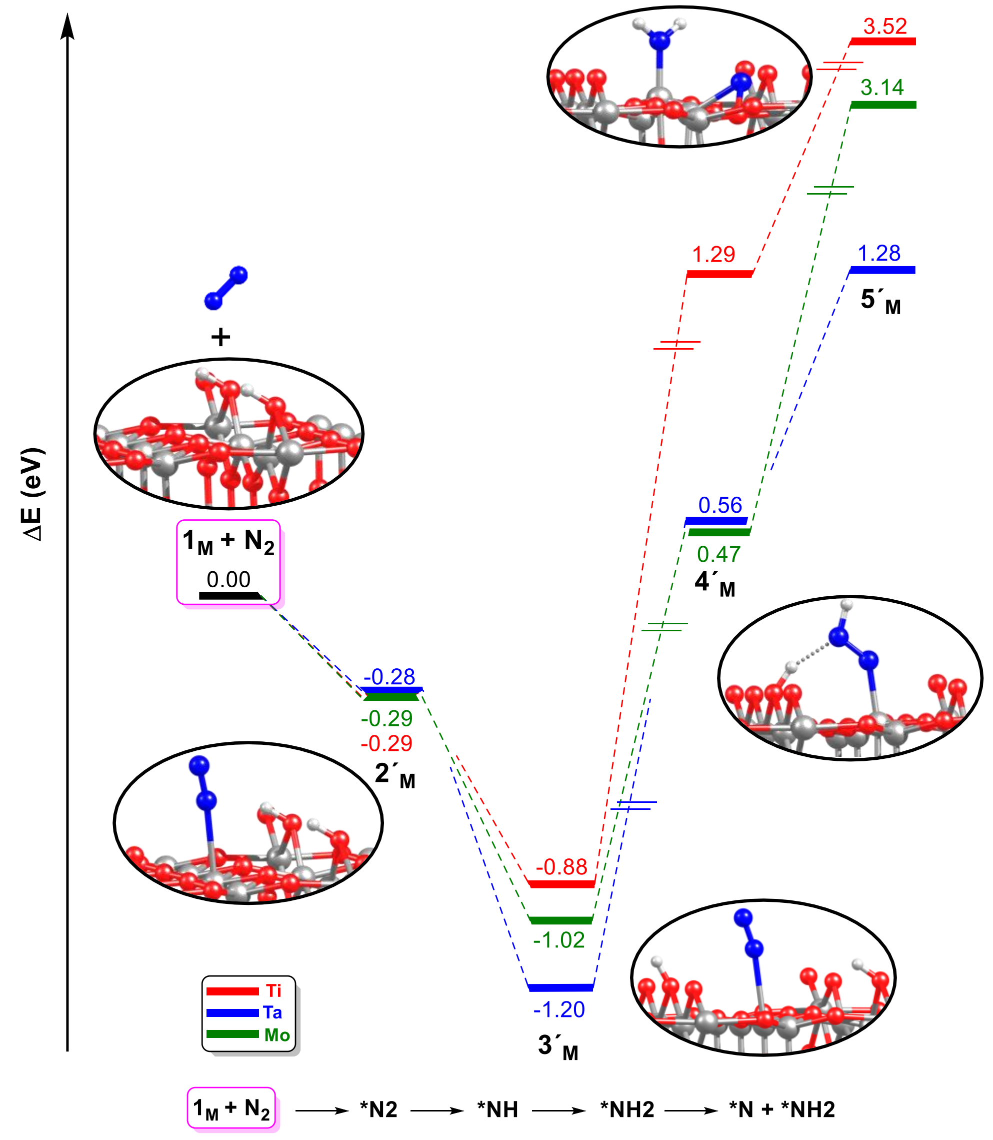

Figure S4. Energy profiles (in eV) of the N_2 adsorption and activation over Ti_{5c} site with doped M_{6c} (M= Ti, Mo, Ta) of the hydroxylated rutile TiO_2 (110) surface with an O_{2c} vacancy defect. The origin of energies is the bare surface (**1**$_{\mathbf{M}}$) and N_2.

Table S2. Relevant distances (in Å) of all intermediates involved in the N_2 adsorption and activation over Ti_{5c} site with doped M_{6c} (M= Ti, Mo, Ta) of the hydroxylated rutile TiO_2 (110) surface with an O_{2c} vacancy defect. See Figure S4 for energies.

	Metal	*N-N	Ti_{5c}-*N	N···H	Ti_{5c}-*NH2
	**Ti**	1.11	2.60		
**2´M**	**Mo**	1.11	2.59		
	**Ta**	1.11	2.63		
	**Ti**	1.11	2.60		
**3´M**	**Mo**	1.11	2.61		
	**Ta**	1.11	2.67		
	**Ti**	1.22	2.02	1.80	
**4´M**	**Mo**	1.21	2.01	1.85	
	**Ta**	1.22	2.01	1.81	
	**Ti**	4.35	1.93		1.88
**5´M**	**Mo**	2.81	1.75		1.87
	**Ta**	1.41	1.80		2.23

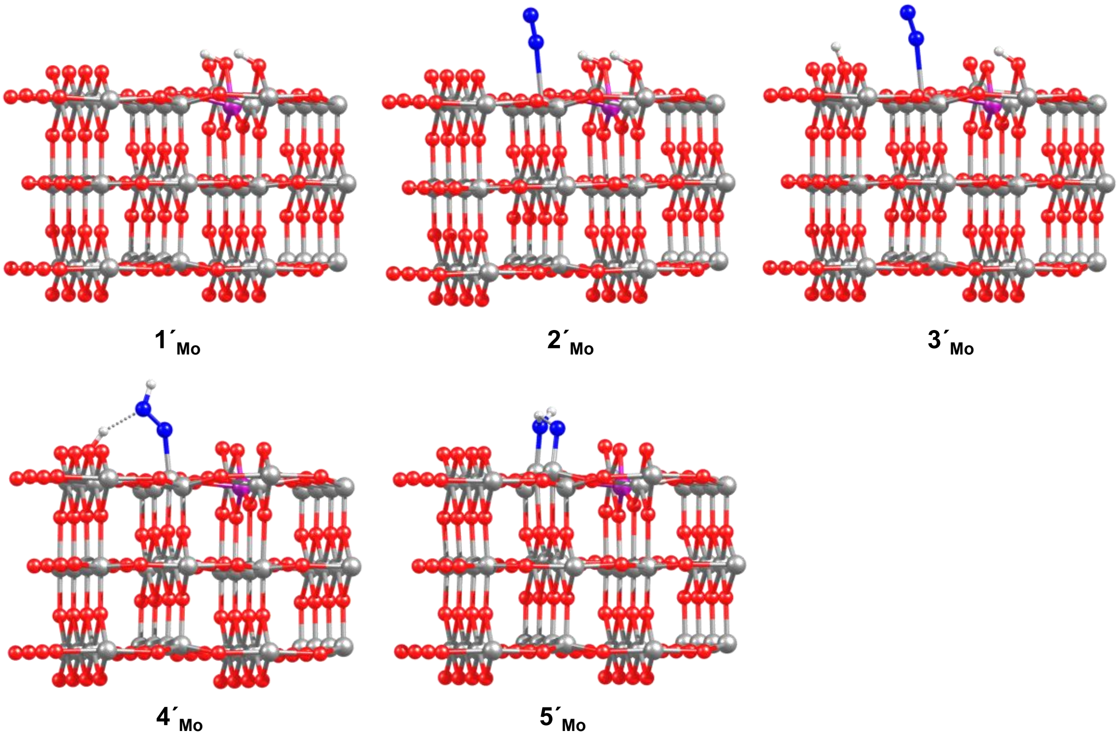

Figure S5. Optimized structures of the intermediates involved in the N_2 adsorption and activation over Ti_{5c} site with doped Mo_{6c} of the hydroxylated rutile TiO_2 (110) surface with an O_{2c} vacancy defect.

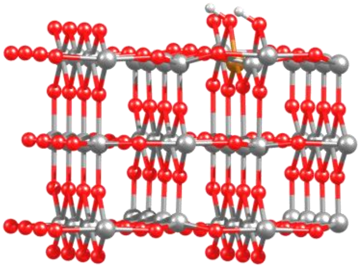

$1'_{Ta}$

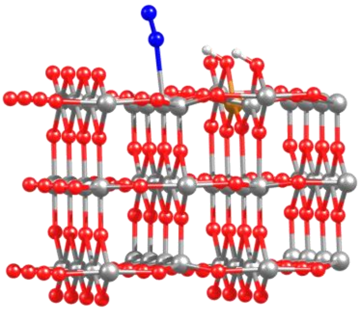

$2'_{Ta}$

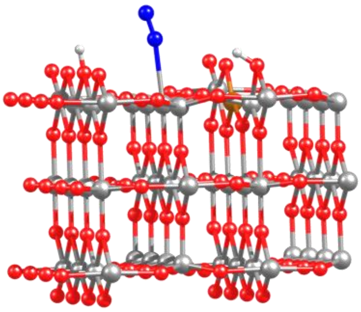

$3'_{Ta}$

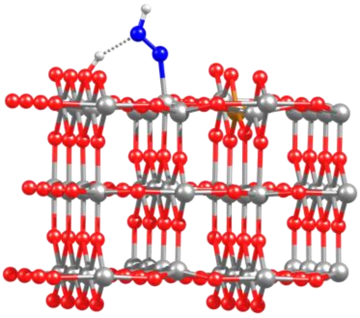

$4'_{Ta}$

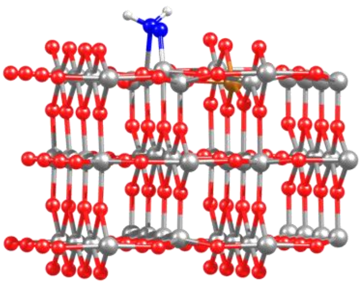

$5'_{Ta}$

Figure S6. Optimized structures of the intermediates involved in the N_2 adsorption and activation over Ti_{5c} site with doped Ta_{6c} of the hydroxylated rutile TiO_2 (110) surface with an O_{2c} vacancy defect.

Table S3. Relevant distances (in Å) of all intermediates involved in the N_2 adsorption and activation over M_{6c} (M= Ti, Mo, Ta) sites of the hydroxylated rutile TiO_2 (110) surface with an O_{2c} vacancy defect. The values corresponding to the two *k* point sets are shown. See Figure 3 and S10 for energies.

				**1 x 1 x 1 *k* point mesh**			
	**Metal**	***N-N**	**M1-*N**	**Ti2-*N**	**Ti_{5c}-*N**	**M1-*N**	**Ti2-*N**
	**Ti**	1.11	2.93	2.93			
**6_M**	**Mo**	1.15	1.98	2.70			
	**Ta**	1.11	2.84	2.96			
	**Ti**	1.22	2.14	2.25			
**7_M**	**Mo**	1.23	1.84	2.56			
	**Ta**	1.23	2.16	2.30			
	**Ti**	1.23	2.22	2.22			
**8_M**	**Mo**	1.29	1.83	2.46			
	**Ta**	1.25	2.24	2.18			
	**Ti**	2.73			1.89	2.07	3.00
**9_M**	**Mo**	3.45			1.88	1.73	2.14
	**Ta**	3.46			1.87	1.98	1.92
				**3 x 3 x 1 *k* point mesh**			
	**Metal**	***N-N**	**M1-*N**	**Ti2-*N**	**Ti_{5c}-*N**	**M1-*N**	**Ti2-*N**
	**Ti**	1.11	2.90	2.90			
**6_M**	**Mo**	1.15	1.98	2.71			
	**Ta**	1.11	2.79	2.93			
	**Ti**	1.22	2.15	2.23			
**7_M**	**Mo**	1.23	1.84	2.55			
	**Ta**	1.23	2.17	2.30			
	**Ti**	1.24	2.21	2.20			
**8_M**	**Mo**	1.29	1.83	2.45			
	**Ta**	1.26	2.20	2.16			
	**Ti**	2.72			1.89	2.06	2.99
**9_M**	**Mo**	3.45			1.88	1.73	2.13
	**Ta**	3.44			1.88	1.98	1.93

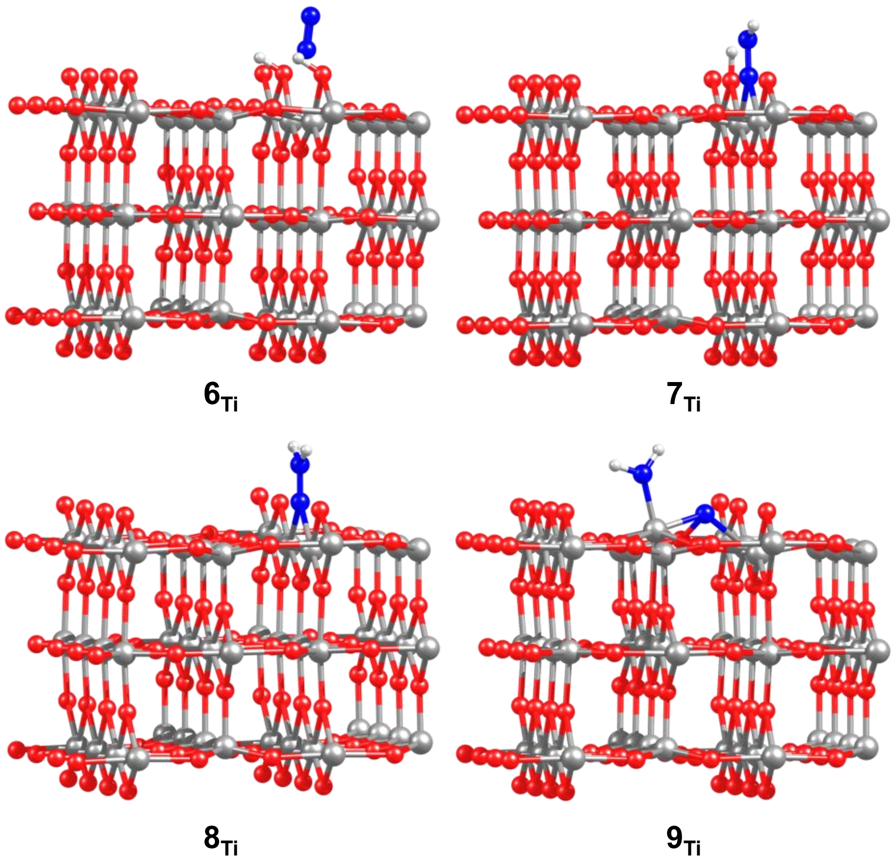

Figure S7. Optimized structures of the intermediates involved in the N_2 adsorption and activation over Ti_{6c} site of the hydroxylated rutile TiO_2 (110) surface with an O_{2c} vacancy defect.

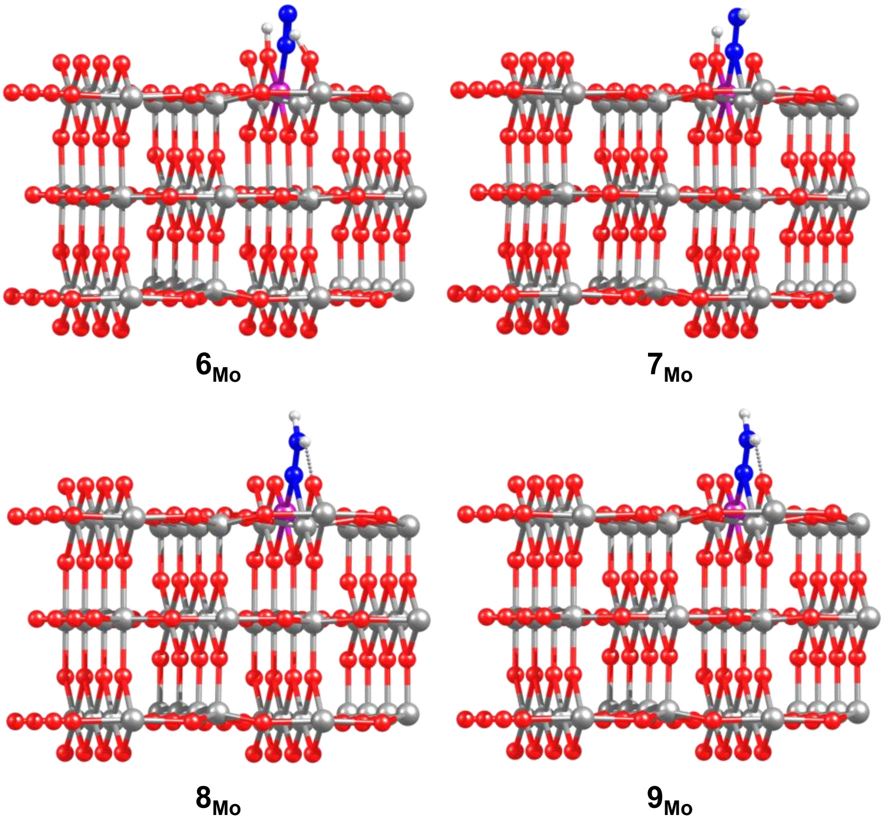

Figure S8. Optimized structures of the intermediates involved in the N_2 adsorption and activation over Mo_{6c} site of the hydroxylated rutile TiO_2 (110) surface with an O_{2c} vacancy defect.

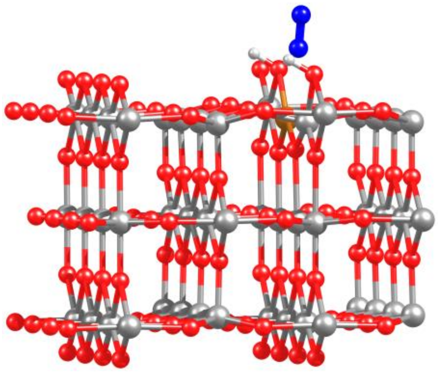

6_{Ta}

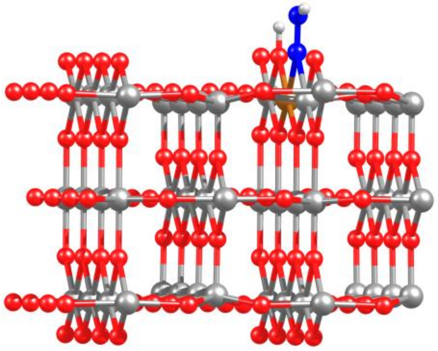

7_{Ta}

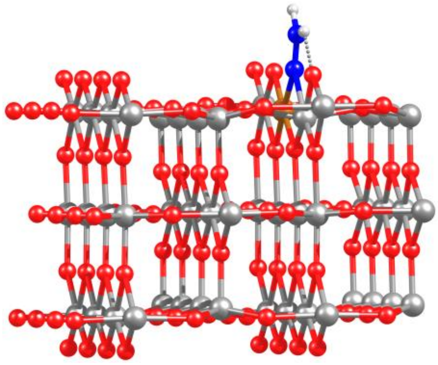

8_{Ta}

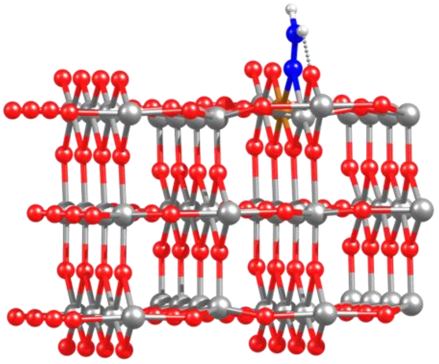

9_{Ta}

Figure S9. Optimized structures of the intermediates involved in the N_2 adsorption and activation over Ta_{6c} site of the hydroxylated rutile TiO_2 (110) surface with an O_{2c} vacancy defect.

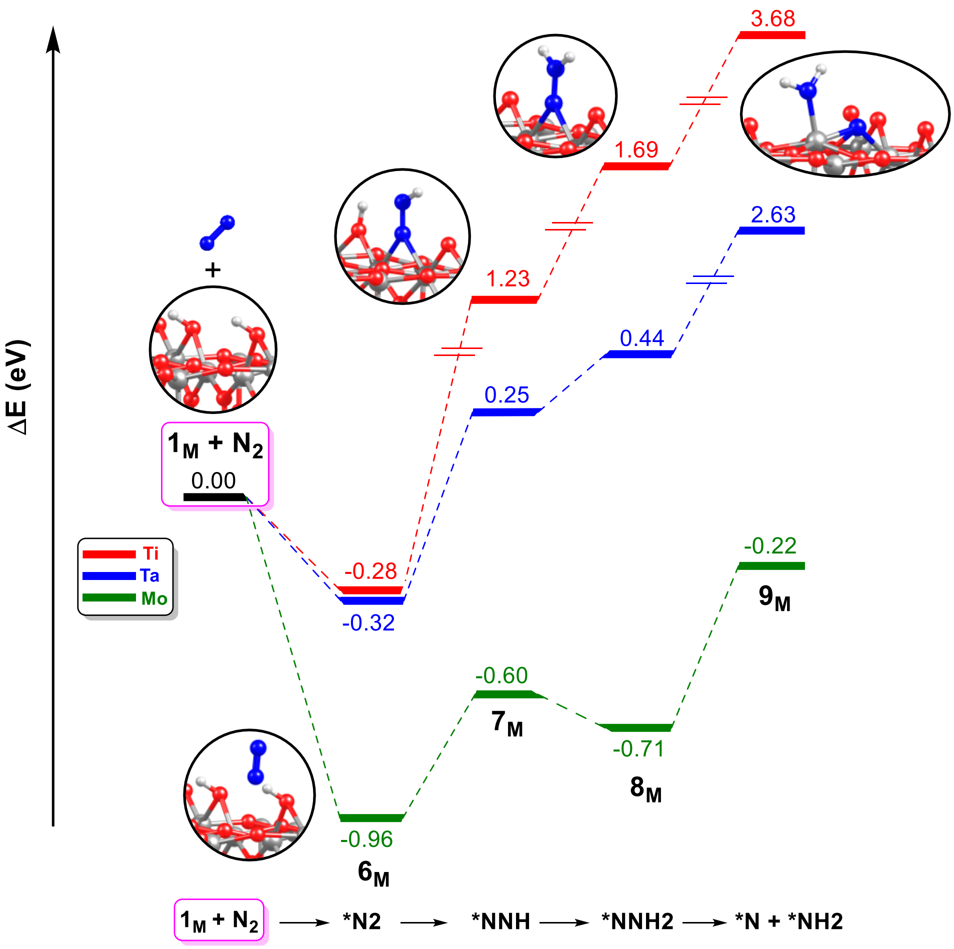

Figure S10. Energy profiles (in eV) of the N_2 adsorption, hydrogenation, and activation over M_{6c} sites (M= Ti, Mo, Ta) of the hydroxylated rutile TiO_2 (110) surface with an O_{2c} vacancy defect, using a 3 x 3 x 1 *k* point mesh. The origin of energies is the bare surface (**1$_M$**) and N_2.

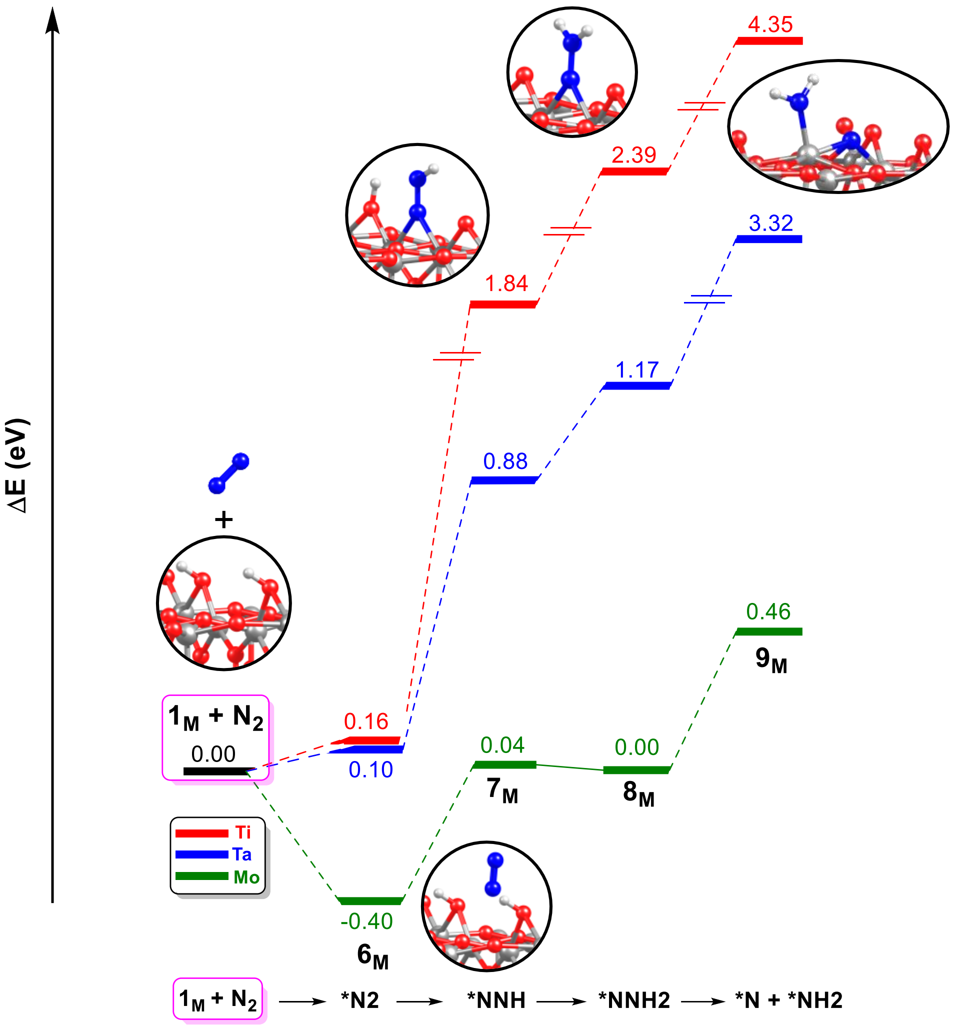

Figure S11. Gibbs energy profiles (in eV) at 298 K of the N_2 adsorption, hydrogenation, and activation over M_{6c} sites (M= Ti, Mo, Ta) of the hydroxylated rutile TiO_2 (110) surface with an O_{2c} vacancy defect, using a 1 x 1 x 1 *k* point mesh. The origin of energies is the bare surface (**1**$_{\mathbf{M}}$) and N_2.

Table S4. Relative energies (in eV) of the N_2 adsorption, hydrogenation, and activation over M_{6c} sites (M= Ti, Mo, Ta) of the hydroxylated rutile TiO_2 (110) surface with an O_{2c} vacancy defect with and without spin polarization. The origin of energies is the bare surface (**1_M**) and N_2.

**Metal →**	**Ti**		**Ta**		**Mo**	
**Intermediate ↓**	**Spin-polarized**	**Non-spin-polarized**	**Spin-polarized**	**Non-spin-polarized**	**Spin-polarized**	**Non-spin-polarized**
**6_M**	-0.29	-0.28	-0.36	-0.31	-0.66	-0.87
**7_M**	1.20	1.37	0.38	0.40	-0.35	-0.46
**8_M**	1.68	1.82	0.38	0.61	-0.45	-0.54
**9_M**	4.69	3.84	2.56	2.86	0.26	-0.01

Table S5. Relevant distances (in Å) of all intermediates involved in the N_2 activation over M_{6c} (M= Ti, Mo, Ta) sites of the hydroxylated rutile TiO_2 (110) surface with an O_{2c} vacancy defect, starting from **10_M** (*NNH2 species) following direct *N-N bond cleavage to *NH2 and *N. See Figure 4 for energies.

	**Metal**	***N-N**	**M1-*N**	**Ti2-*N**	**Ti5c-*N**	**M1-*N**	**Ti2-*N**
	**Ti**	1.30	2.04	2.04			
**10_M**	**Mo**	1.31	1.81	2.53			
	**Ta**	1.36	1.85	2.45			
	**Ti**	3.26			1.88	1.83	1.83
**11_M**	**Mo**	3.45			1.89	1.71	2.27
	**Ta**	3.43			1.88	1.90	1.82
	**Ti**	3.48			1.89	1.97	1.89
**12_M**	**Mo**	3.47			1.90	1.78	2.42
	**Ta**	3.51			1.87	1.90	2.11
	**Ti**	3.40			1.90	2.13	2.12
**13_M**	**Mo**	3.26			1.91	2.13	2.18
	**Ta**	3.36			1.90	2.16	2.19

Table S6. Relevant distances (in Å) of all intermediates involved in the N_2 activation over M_{6c} (M= Ti, Mo, Ta) sites of the hydroxylated rutile TiO_2 (110) surface with an O_{2c} vacancy defect, starting **10_M** (*NNH2 species) following first hydrogenation to *NNH3 and then *N-N bond cleavage to *NH3 and *N. See Figure 4 for energies.

	Metal	*N-N	M1-*N	Ti2-*N	Ti5c-*N	M1-*N	Ti2-*N
	**Ti**	1.41	1.97	2.08			
**14_M**	**Mo≠**	1.30	1.84	2.37			
	**Ta≠**	1.38	1.86	2.49			
	**Ti**	3.00			2.19	1.82	1.85
**15_M**	**Mo**	3.26			2.23	1.72	2.24
	**Ta**	3.02			2.20	1.88	1.87
	**Ti**	3.82			2.20	1.94	1.91
**16_M**	**Mo**	3.83			2.22	1.80	2.31
	**Ta**	3.80			2.21	1.90	2.09

≠No *NNH3 formed. H transferred to neighbor O.

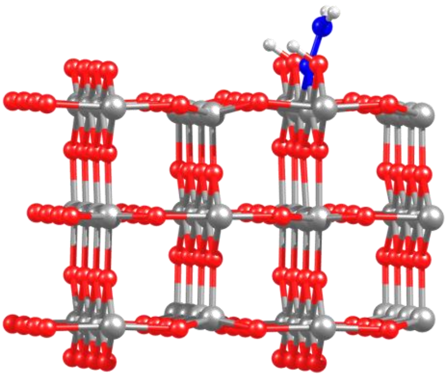

10_{Ti}

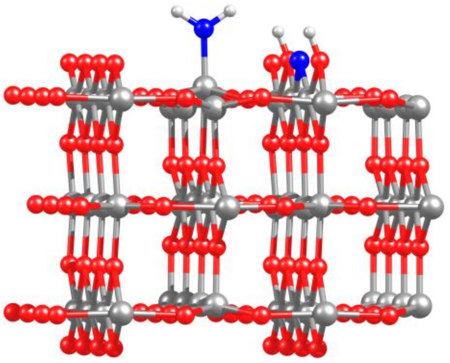

11_{Ti}

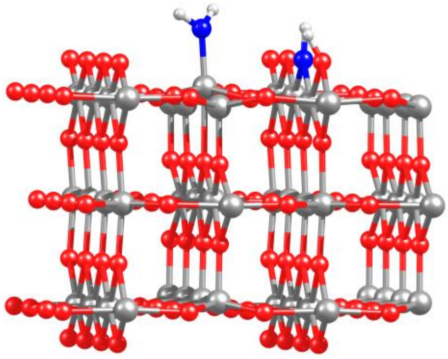

12_{Ti}

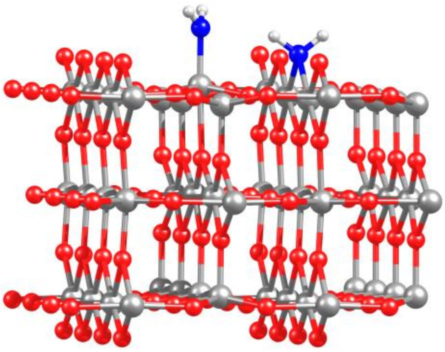

13_{Ti}

Figure S12. Optimized structures of the intermediates involved in the N_2 activation over Ti_{6c} site of the hydroxylated rutile TiO_2 (110) surface with an O_{2c} vacancy defect, starting from **10_{Ti}** (*NNH2 species) following direct N-N bond cleavage to *NH2 and *N.

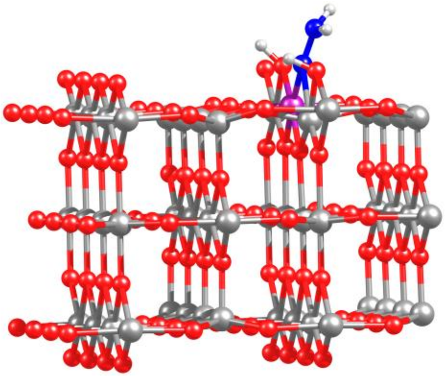

$\mathbf{10_{Mo}}$

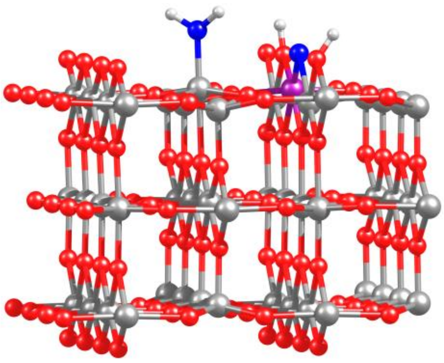

$\mathbf{11_{Mo}}$

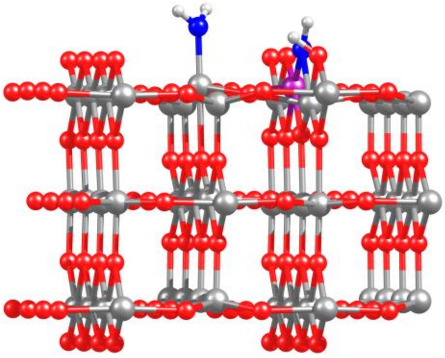

$\mathbf{12_{Mo}}$

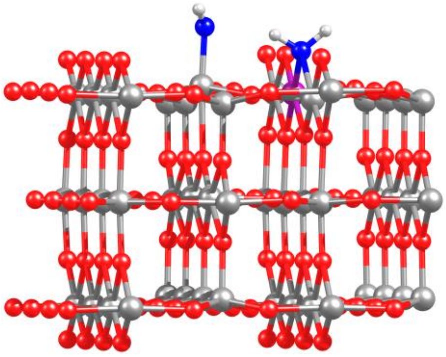

$\mathbf{13_{Mo}}$

Figure S13. Optimized structures of the intermediates involved in the N_2 activation over Mo_{6c} site of the O vacant sites of the rutile TiO_2 (110) surface with an O_{2c} vacancy defect, starting from $\mathbf{10_{Mo}}$ (*NNH2 species) following direct N-N bond cleavage to *NH2 and *N.

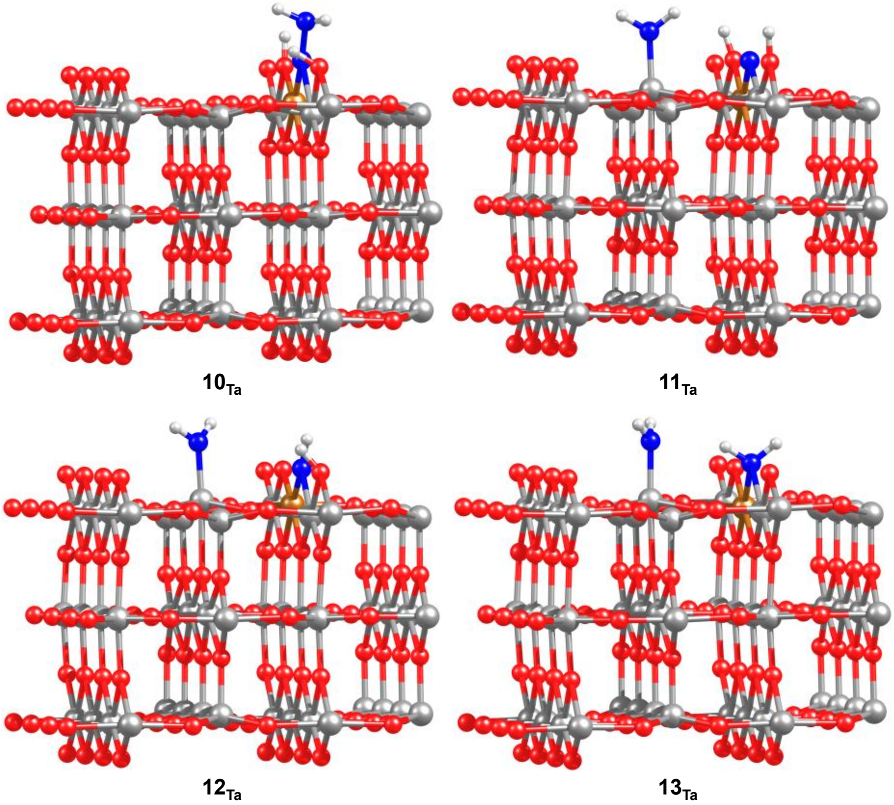

Figure S14. Optimized structures of the intermediates involved in the N_2 activation over Ta_{6c} site of the O_{2c} vacant sites of the rutile TiO_2 (110) surface, starting from **10_{Ta}** (*NNH2 species) following direct N-N bond cleavage to *NH2 and *N.

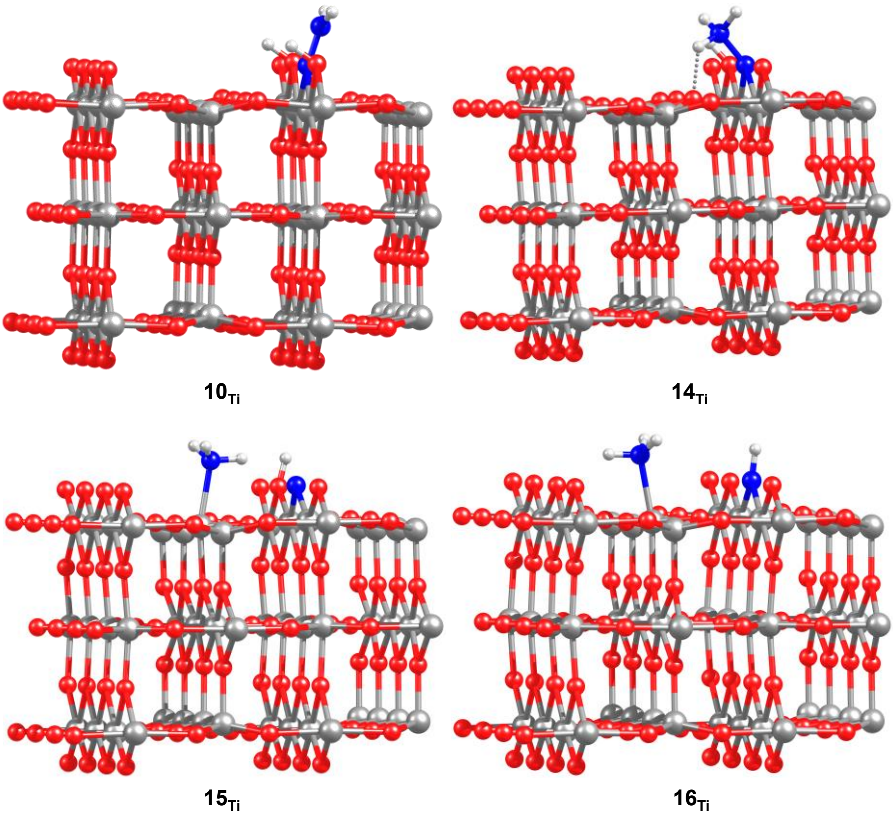

Figure S15. Optimized structures of the intermediates involved in the N_2 activation over Ti_{6c} site of the hydroxylated rutile TiO_2 (110) surface with an O_{2c} vacancy defect, starting from $\mathbf{10_{Ti}}$ (*NNH2 species) following first hydrogenation to *NNH3 and then *N-N bond cleavage to *NH3 and *N.

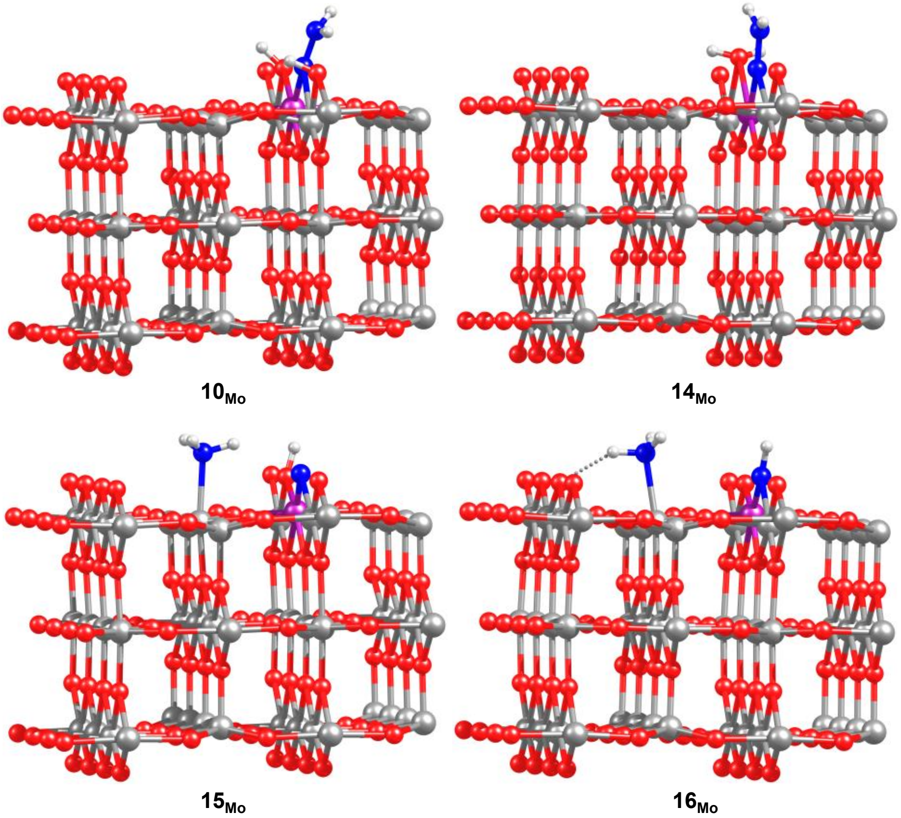

Figure S16. Optimized structures of the intermediates involved in the N_2 activation over Mo_{6c} site of the hydroxylated rutile TiO_2 (110) surface with an O_{2c} vacancy defect, starting from $\mathbf{10_{Mo}}$ (*NNH2 species) following first hydrogenation to *NNH3 and then *N-N bond cleavage to *NH3 and *N.

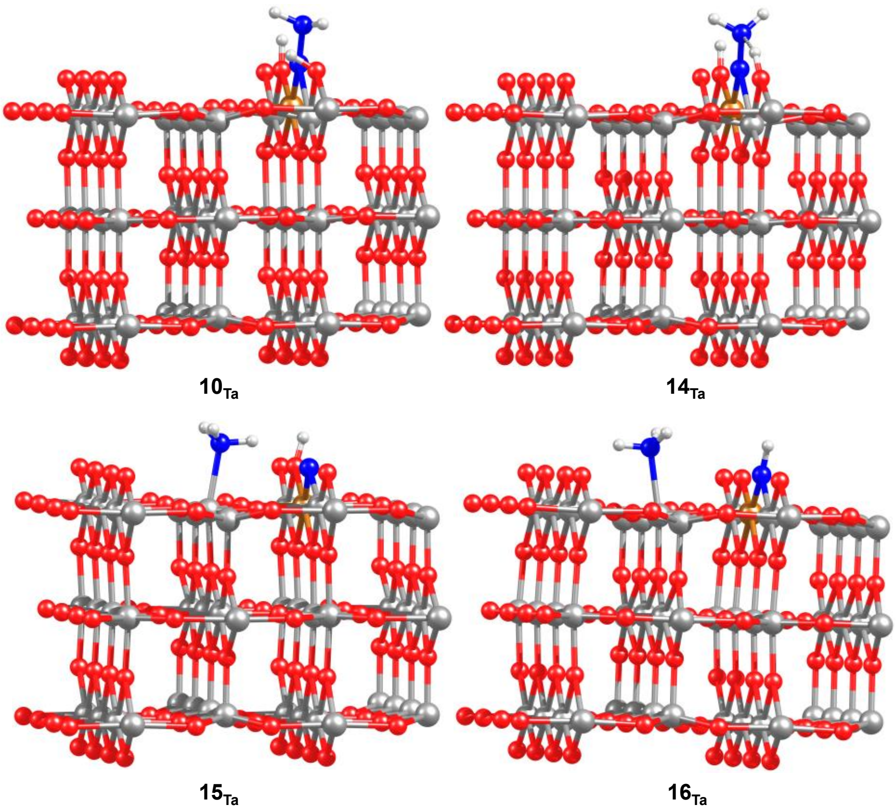

Figure S17. Optimized structures of the intermediates involved in the N_2 activation over Ta_{6c} site of the hydroxylated rutile TiO_2 (110) surface with an O_{2c} vacancy defect, starting from $\mathbf{10_{Ta}}$ (*NNH2 species) following first hydrogenation to *NNH3 and then *N-N bond cleavage to *NH3 and *N.

Table S7. Relevant distances (in Å) of all intermediates involved in the N_2 hydrogenation to NH_3 over M_{6c} (M= Ti, Mo, Ta) sites of the hydroxylated rutile TiO_2 (110) surface with an O_{2c} vacancy defect. See Figure 5 for energies.

	Metal	Ti5c-*N	M1-*N	Ti2-*N
	**Ti**	1.92	2.15	2.15
**17_M**	**Mo**	1.94	2.13	2.24
	**Ta**	1.92	2.16	2.23
	**Ti**	2.22	2.12	1.83
**18_M**	**Mo**	2.23	1.93	1.98
	**Ta**	2.24	1.85	2.29
	**Ti**	1.93	2.57	2.62
**19_M**	**Mo**	1.95	2.43	2.88
	**Ta**	1.95	2.44	2.90
	**Ti**	2.24	2.17	2.12
**20_M**	**Mo**	2.25	2.07	2.25
	**Ta**	2.25	2.20	2.18
	**Ti**	2.24	2.66	2.60
**21_M**	**Mo**	2.26	2.48	2.88
	**Ta**	2.26	2.48	2.91

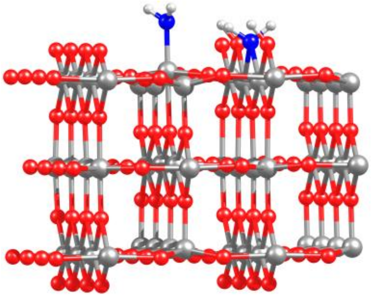

17_{Ti}

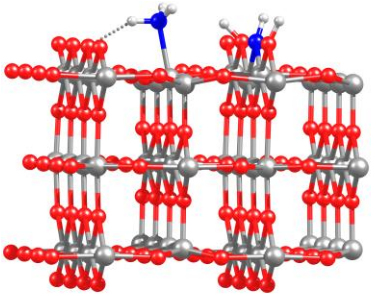

18_{Ti}

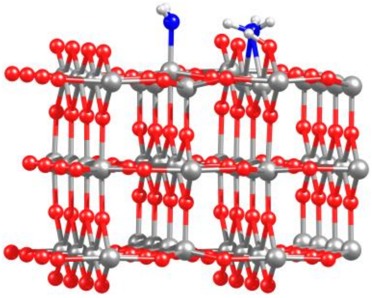

19_{Ti}

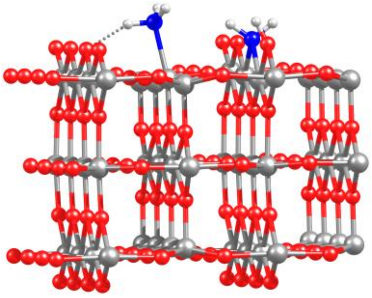

20_{Ti}

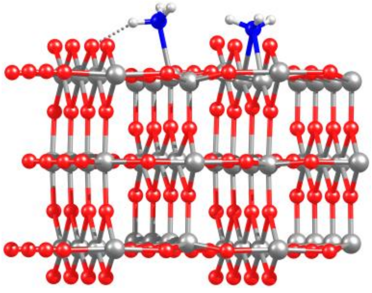

21_{Ti}

Figure S18. Optimized structures of the intermediates involved in the N_2 hydrogenation to NH_3 over Ti_{6c} site of the hydroxylated rutile TiO_2 (110) surface with an O_{2c} vacancy defect.

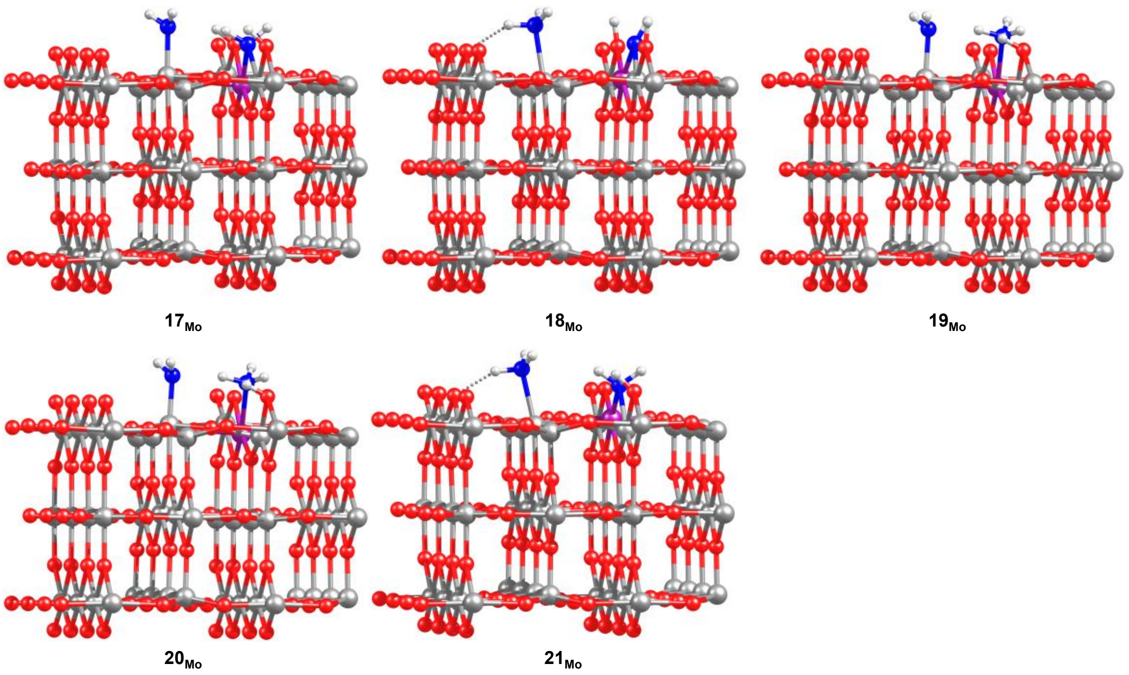

Figure S19. Optimized structures of the intermediates involved in the N_2 hydrogenation to NH_3 over Mo_{6c} site of the hydroxylated rutile TiO_2 (110) surface with an O_{2c} vacancy defect.

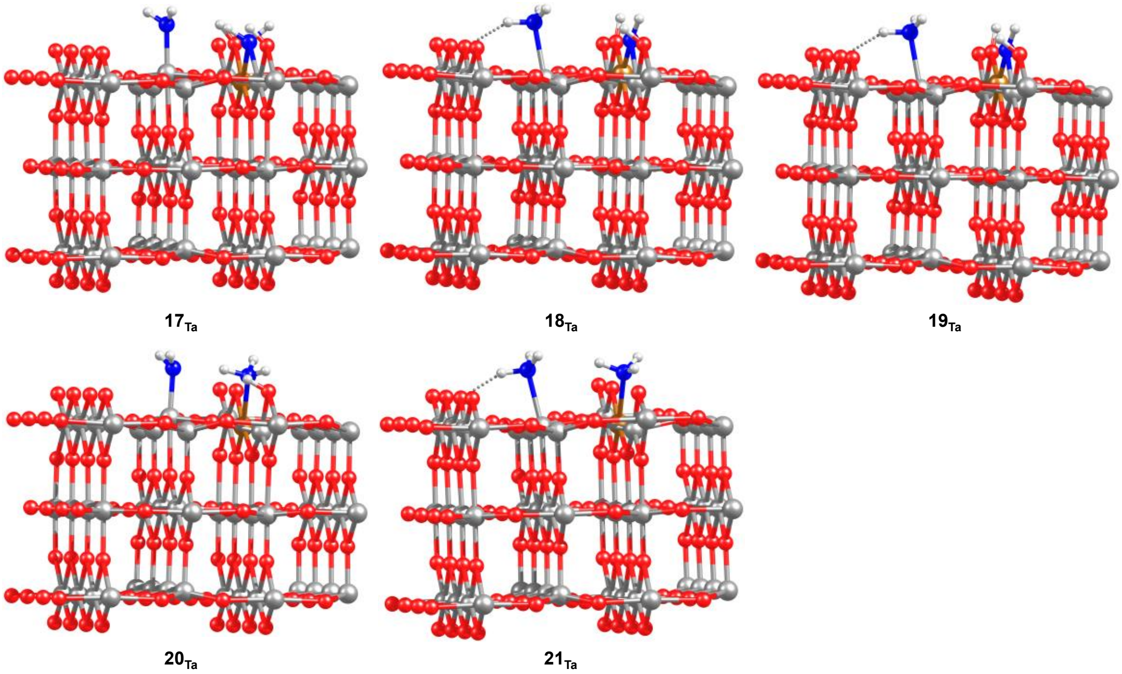

Figure S20. Optimized structures of the intermediates involved in the N_2 hydrogenation to NH_3 over Ta_{6c} site of the hydroxylated rutile TiO_2 (110) surface with an O_{2c} vacancy defect.

Table S8. Relevant distances (in Å) of all intermediates involved in the N_2 adsorption and activation over M_{6c} (M= Ti, Mo, Ta) of the hydroxylated rutile TiO_2 (110) surface with an O_{2c} vacancy defect with 2 doping atoms in the vacancy. See Figure 6 for energies.

	Metal	*N-N	M1-*N	M2-*N	Ti_{5c}-*N
**6_{2M}**	**Ti**	1.11	2.93	2.93	
	**Mo**	1.17	1.93	2.25	
	**Ta**	1.19	2.13	2.21$^{\neq}$	
**7_{2M}**	**Ti**	1.22	2.14	2.25	
	**Mo**	1.23	1.85	2.69	
	**Ta**	1.28	1.95	2.12$^{\neq}$	
**8_{2M}**	**Ti**	1.23	2.22	2.22	
	**Mo**	1.30	1.97	2.13	
	**Ta**	1.30	2.13	2.14	
**9_{2M}**	**Ti**	2.73	2.07	3.00	1.89
	**Mo**	3.46	1.78	1.98	1.88
	**Ta**	3.24	1.91	1.91	1.87

$^{\neq}$Ta-*N bond with the other *N atom.

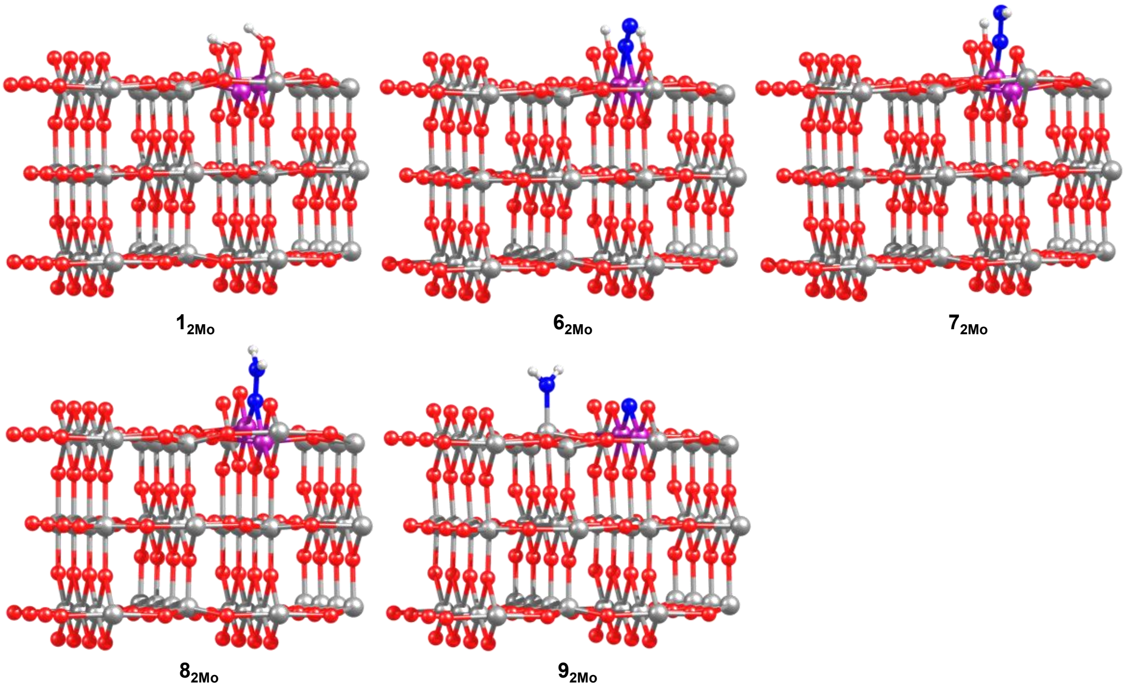

Figure S21. Optimized structures of the intermediates involved in the N_2 adsorption and activation over Mo_{6c} site of the hydroxylated rutile TiO_2 (110) surface with an O_{2c} vacancy defect with 2 doping atoms in the vacancy.

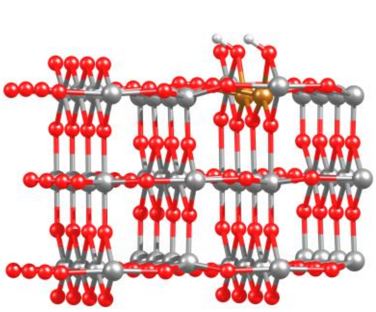

$\mathbf{1_{2Ta}}$

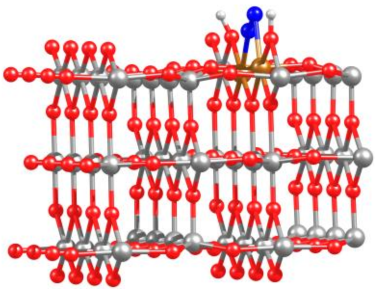

$\mathbf{6_{2Ta}}$

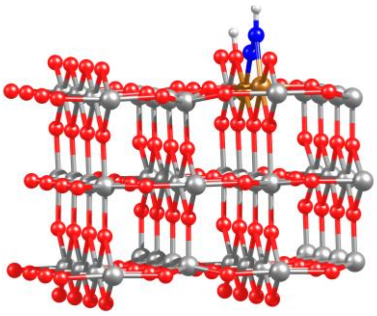

$\mathbf{7_{2Ta}}$

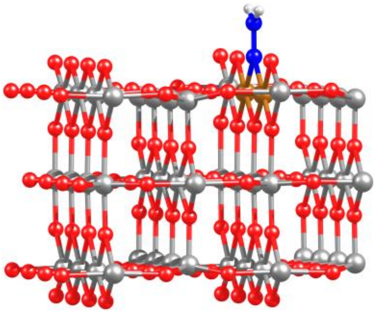

$\mathbf{8_{2Ta}}$

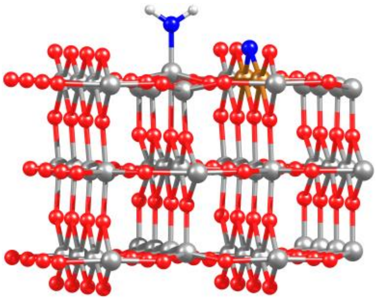

$\mathbf{9_{2Ta}}$

Figure S22. Optimized structures of the intermediates involved in the N_2 adsorption and activation over Ta_{6c} site of the hydroxylated rutile TiO_2 (110) surface with an O_{2c} vacancy defect with 2 doping atoms in the vacancy.

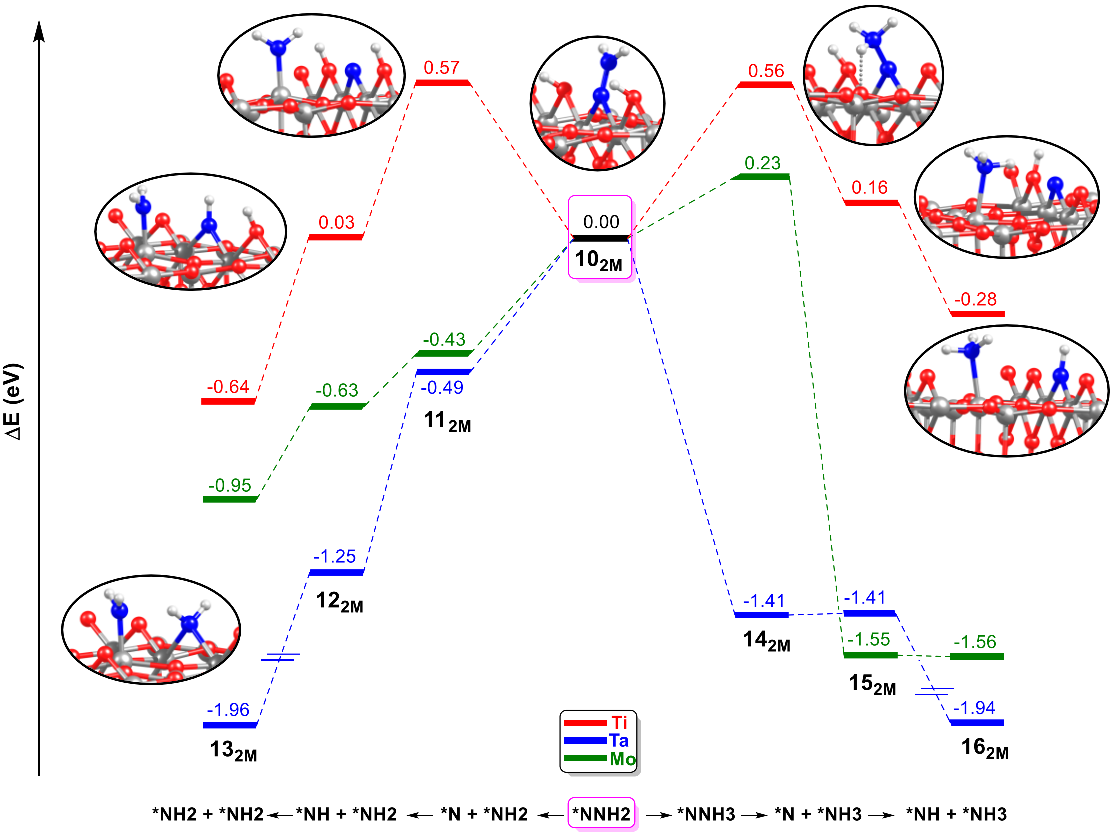

Figure S23. Energy profile (in eV) of the N_2 activation over M_{6c} (M= Ti, Mo, Ta) sites of the hydroxylated rutile TiO_2 (110) surface with an O_{2c} vacancy defect with 2 doping atoms in the vacancy, starting from $\mathbf{10_{2M}}$ (*NNH2 species), following two mechanisms: direct N-N bond cleavage to *NH2 and *N (left) and first hydrogenation to *NNH3 and then N-N bond cleavage to *NH3 and *N (right). The origin of energies is the intermediate $\mathbf{10_{2M}}$.

Table S9. Relevant distances (in Å) of all intermediates involved in the N_2 activation over M_{6c} (M= Ti, Mo, Ta) sites of the hydroxylated rutile TiO_2 (110) surface with an O_{2c} vacancy defect with 2 doping atoms in the vacancy, starting from **10_{2M}** (*NNH2 species), following direct N-N bond cleavage to *NH2 and *N. See Figure S23 for energies.

	Metal	*N-N	M1-*N	M2-*N	Ti5c-*N
	**Ti**	1.30	2.04	2.04	
**10_{2M}**	**Mo**	1.31	1.98	1.98	
	**Ta**	1.39	1.88	2.46	
	**Ti**	3.26	1.83	1.83	1.88
**11_{2M}**	**Mo**	3.53	1.74	2.12	1.89
	**Ta**	3.51	1.90	1.90	1.88
	**Ti**	3.48	1.97	1.89	1.89
**12_{2M}**	**Mo**	3.43	1.98	1.96	1.91
	**Ta**	3.52	2.14	1.92	1.89
	**Ti**	3.40	2.13	2.12	1.90
**13_{2M}**	**Mo**	3.23	2.12	2.24	1.92
	**Ta**	3.33	2.21	2.21	1.91

Table S10. Relevant distances (in Å) of all intermediates involved in the N_2 activation over M_{6c} (M= Ti, Mo, Ta) sites of the hydroxylated rutile TiO_2 (110) surface with an O_{2c} vacancy defect with 2 doping atoms in the vacancy, starting from **10_{2M}** (*NNH2 species), following first hydrogenation to *NNH3 and then N-N bond cleavage to *NH3 and *N. See Figure S23 for energies.

	Metal	*N-N	M1-*N	M2-*N	Ti5c-*N
	**Ti**	1.41	1.97	2.08	
**14_{2M}**	**Mo**	1.45	1.95	2.02	
	**Ta**	1.42	2.07	2.16	
	**Ti**	3.00	1.82	1.85	2.19
**15_{2M}**	**Mo**	3.31	1.82	1.92	2.24
	**Ta**	3.07	1.88	1.94	2.22
	**Ti**	3.82	1.94	1.91	2.20
**16_{2M}**	**Mo**	3.81	1.97	1.96	2.23
	**Ta**	3.82	2.03	2.00	2.22

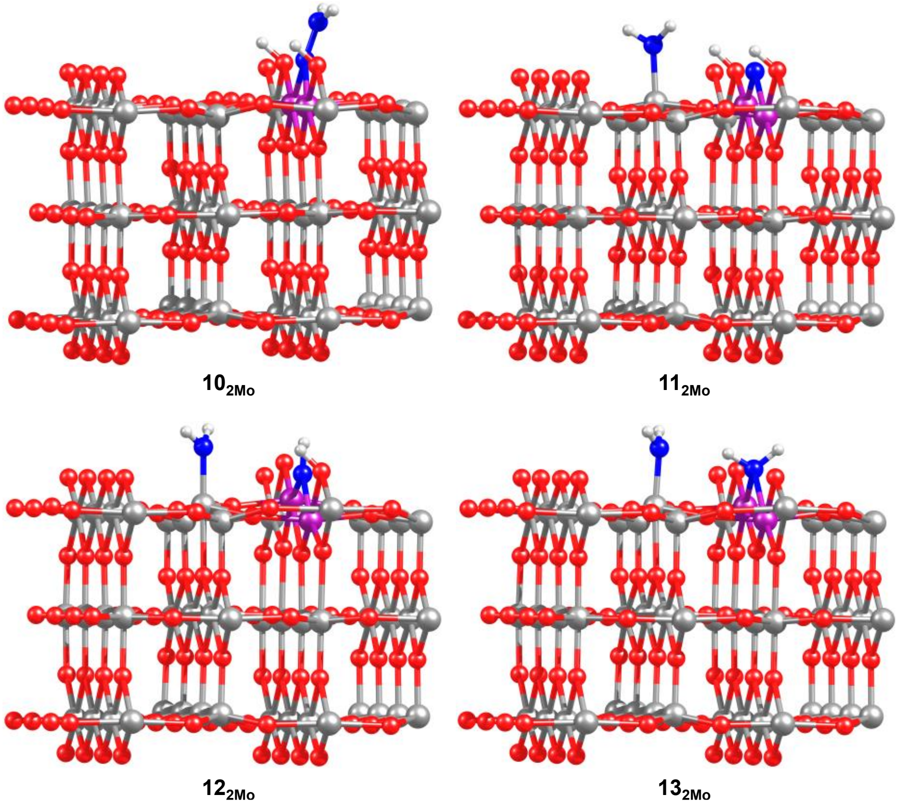

Figure S24. Optimized structures of the intermediates involved in the N_2 activation over Mo_{6c} site of the hydroxylated rutile TiO_2 (110) surface with an O_{2c} vacancy defect with 2 doping atoms in the vacancy, starting from $\mathbf{10_{2Mo}}$ (*NNH2 species) following direct N-N bond cleavage to *NH2 and *N.

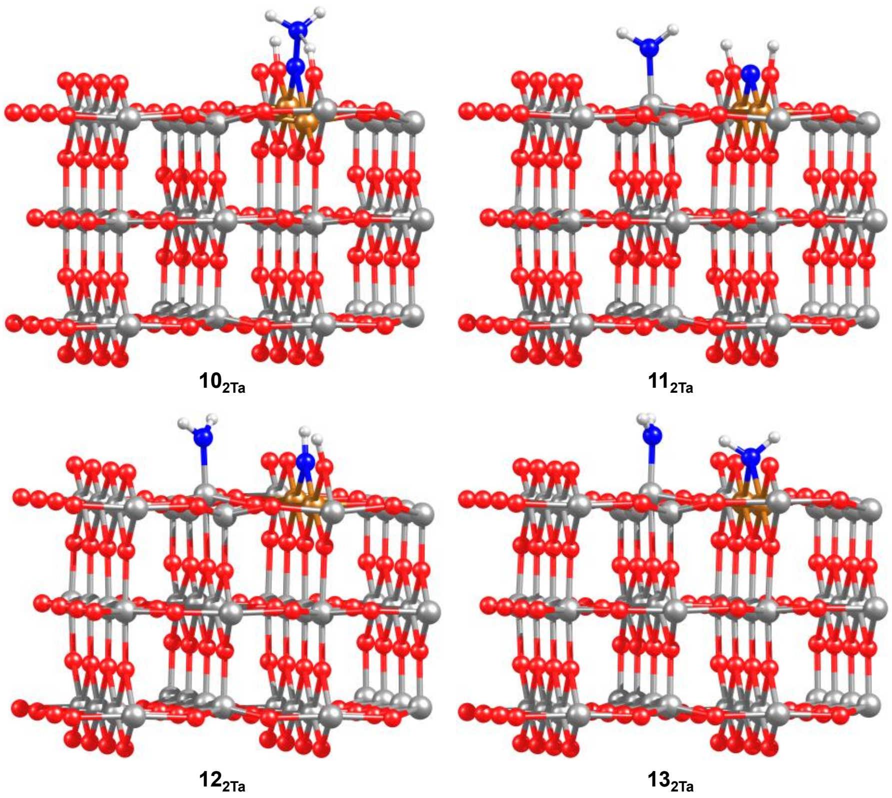

Figure S25. Optimized structures of the intermediates involved in the N_2 activation over Ta_{6c} site of the hydroxylated rutile TiO_2 (110) surface with an O_{2c} vacancy defect with 2 doping atoms in the vacancy, starting from **10_{2Ta}** (*NNH2 species) following direct N-N bond cleavage to *NH2 and *N.

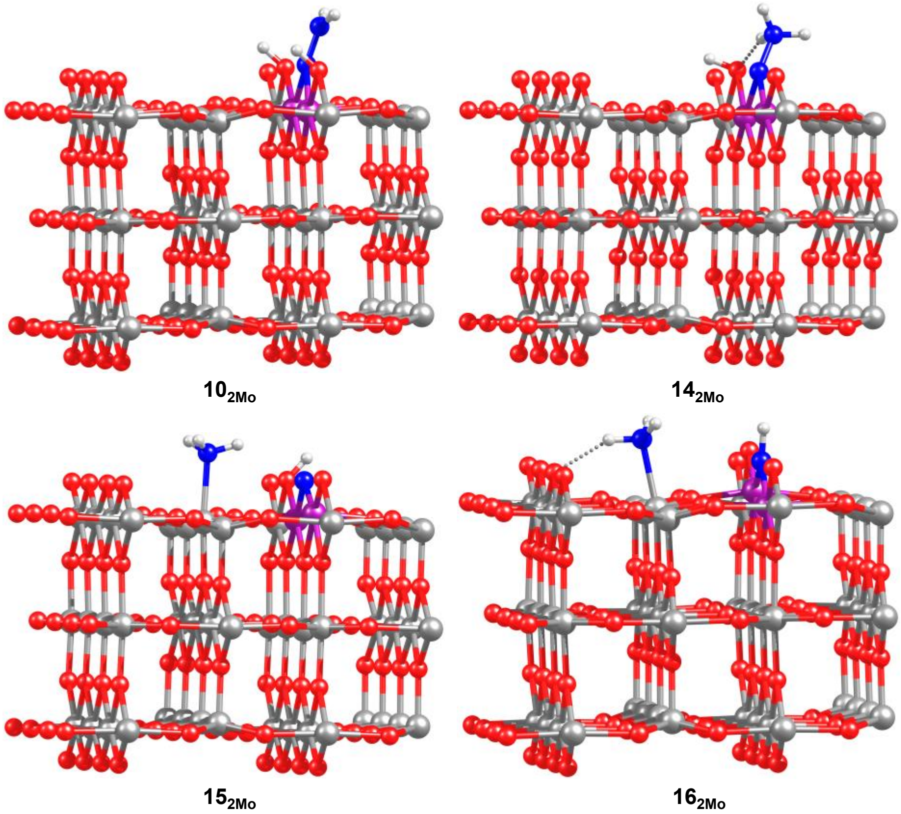

Figure S26. Optimized structures of the intermediates involved in the N_2 activation over Mo_{6c} site of the hydroxylated rutile TiO_2 (110) surface with an O_{2c} vacancy defect with 2 doping atoms in the vacancy, starting from $\mathbf{10_{2Mo}}$ (*NNH2 species) following first hydrogenation to *NNH3 and then N-N bond cleavage to *NH3 and *N.

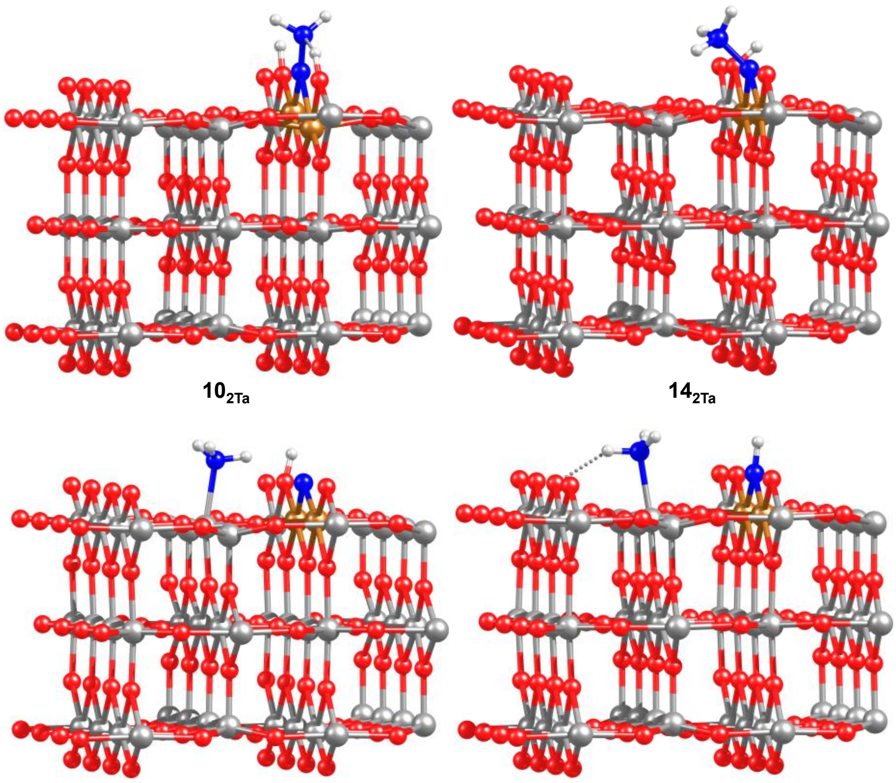

10_{2Ta} **14_{2Ta}**

15_{2Ta} **16_{2Ta}**

Figure S27. Optimized structures of the intermediates involved in the N_2 activation over Ta_{6c} site of the hydroxylated rutile TiO_2 (110) surface with an O_{2c} vacancy defect with 2 doping atoms in the vacancy, starting from **10_{2Ta}** (*NNH2 species) following first hydrogenation to *NNH3 and then N-N bond cleavage to *NH3 and *N.

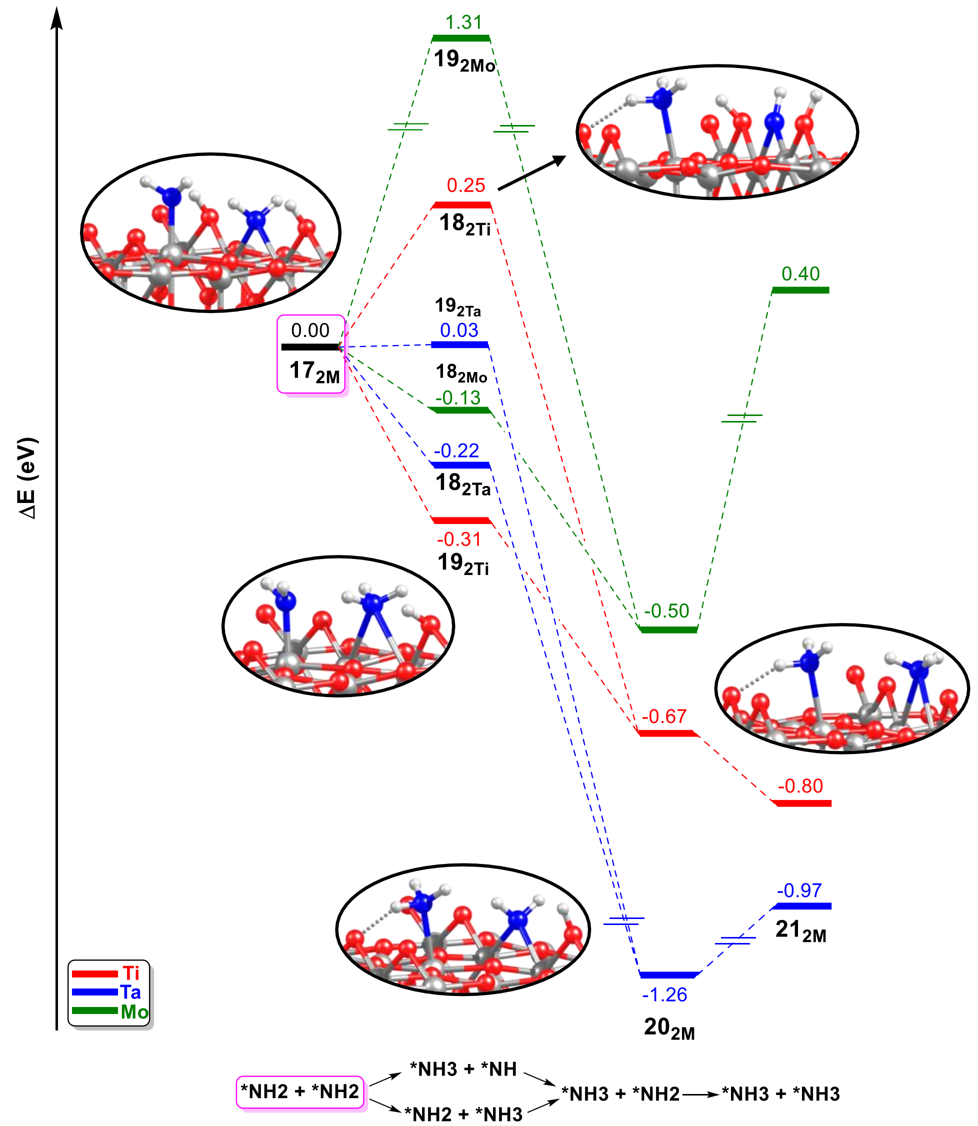

Figure S28. Energy profile (in eV) of the N_2 hydrogenation to NH_3 over M_{6c} (M= Ti, Mo, Ta) sites of the hydroxylated rutile TiO_2 (110) surface with an O_{2c} vacancy defect with 2 doping atoms in the vacancy. The origin of energies is the intermediate **17_{2M}.**

Table S11. Relevant distances (in Å) of all intermediates involved in the N_2 hydrogenation to NH_3 over M_{6c} (M= Ti, Mo, Ta) sites of the hydroxylated rutile TiO_2 (110) surface with an O_{2c} vacancy defect with 2 doping atoms in the vacancy. See Figure S28 for energies.

	Metal	Ti5c-*N	M1-*N	M2-*N
	**Ti**	1.92	2.15	2.15
**17_{2M}**	**Mo**	1.92	2.11	2.10
	**Ta**	1.94	2.23	2.22
	**Ti**	2.22	2.12	1.83
**18_{2M}**	**Mo**	2.24	1.92	2.00
	**Ta**	2.25	2.22	1.91
	**Ti**	1.93	2.57	2.62
**19_{2M}**	**Mo**	1.95	2.46	2.90
	**Ta**	1.96	2.65	2.61
	**Ti**	2.24	2.17	2.12
**20_{2M}**	**Mo**	2.24	2.11	2.12
	**Ta**	2.27	2.26	2.20
	**Ti**	2.24	2.66	2.60
**21_{2M}**	**Mo**	2.27	2.56	2.90
	**Ta**	2.28	2.70	2.67

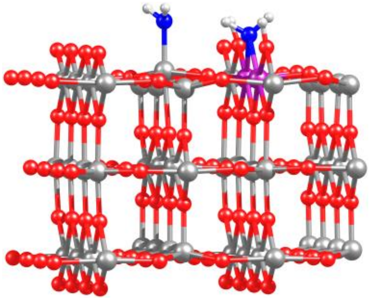

17_{2Mo}

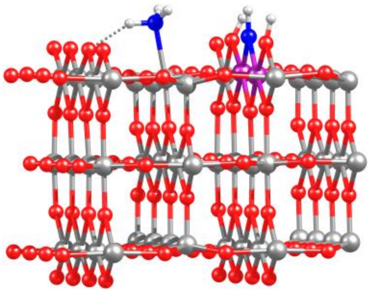

18_{2Mo}

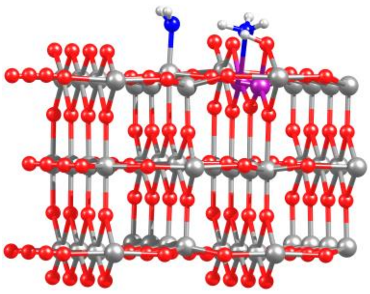

19_{2Mo}

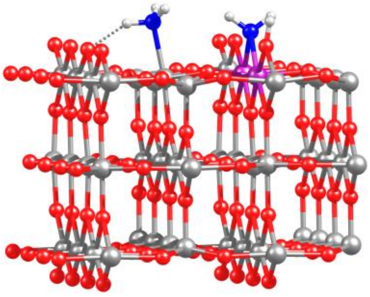

20_{2Mo}

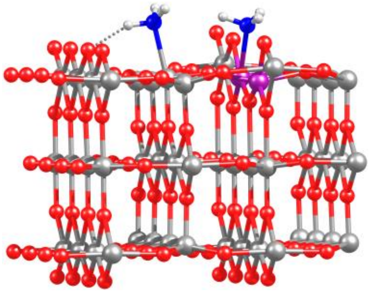

21_{2Mo}

Figure S29. Optimized structures of the intermediates involved in the N_2 hydrogenation to NH_3 over Mo_{6c} site of the hydroxylated rutile TiO_2 (110) surface with an O_{2c} vacancy defect with 2 doping atoms in the vacancy.

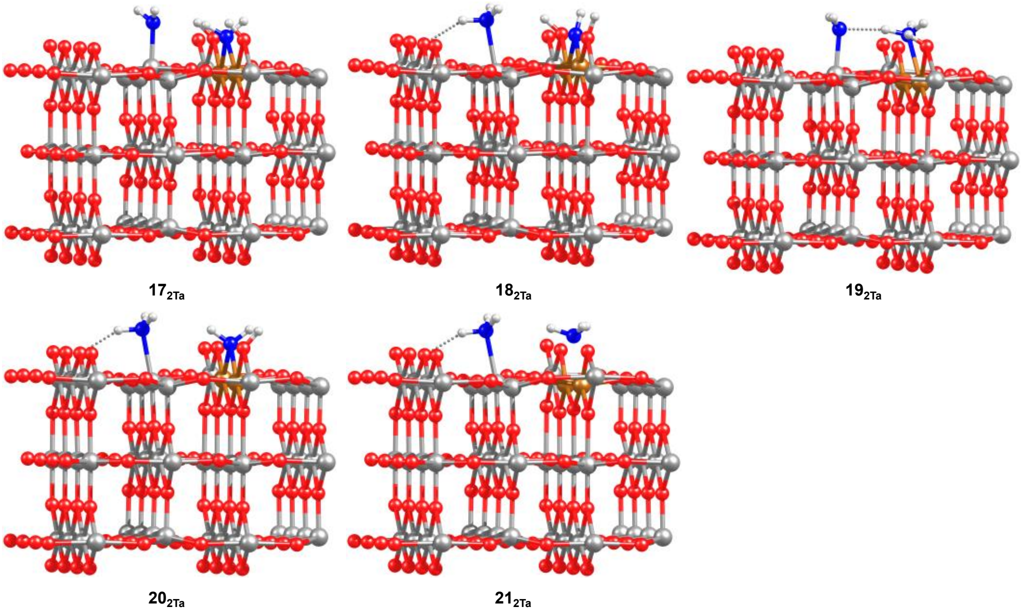

Figure S30. Optimized structures of the intermediates involved in the N_2 hydrogenation to NH_3 over Ta_{6c} site of the hydroxylated rutile TiO_2 (110) surface with an O_{2c} vacancy defect with 2 doping atoms in the vacancy.
